# Radiogenomics: bridging the gap between imaging and genomics for precision oncology

**DOI:** 10.1002/mco2.722

**Published:** 2024-09-09

**Authors:** Wenle He, Wenhui Huang, Lu Zhang, Xuewei Wu, Shuixing Zhang, Bin Zhang

**Affiliations:** ^1^ Department of Radiology The First Affiliated Hospital of Jinan University Guangzhou Guangdong China

**Keywords:** artificial intelligence, oncology, precision medicine, radiogenomics, radiomics

## Abstract

Genomics allows the tracing of origin and evolution of cancer at molecular scale and underpin modern cancer diagnosis and treatment systems. Yet, molecular biomarker‐guided clinical decision‐making encounters major challenges in the realm of individualized medicine, consisting of the invasiveness of procedures and the sampling errors due to high tumor heterogeneity. By contrast, medical imaging enables noninvasive and global characterization of tumors at a low cost. In recent years, radiomics has overcomes the limitations of human visual evaluation by high‐throughput quantitative analysis, enabling the comprehensive utilization of the vast amount of information underlying radiological images. The cross‐scale integration of radiomics and genomics (hereafter radiogenomics) has the enormous potential to enhance cancer decoding and act as a catalyst for digital precision medicine. Herein, we provide a comprehensive overview of the current framework and potential clinical applications of radiogenomics in patient care. We also highlight recent research advances to illustrate how radiogenomics can address common clinical problems in solid tumors such as breast cancer, lung cancer, and glioma. Finally, we analyze existing literature to outline challenges and propose solutions, while also identifying future research pathways. We believe that the perspectives shared in this survey will provide a valuable guide for researchers in the realm of radiogenomics aiming to advance precision oncology.

## Graphical abstract

Radiogenomics is a new tool for precisive oncology by deeply integrating radiomics and genomics. This review deconstructs the current research status of radiogenomics through the multidimensional feature engineering of radiomics and the integration patterns of radiomics and genomics, and takes gliomas, lung cancers, breast cancers, and other common cancers as examples to show the advance of the application of radiogenomics in the diagnosis, treatment, and monitoring of tumors.

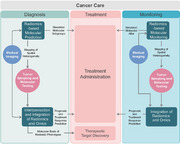

## 1 INTRODUCTION

In 2020, it is estimated that there were approximately 19.3 million new cancer cases and nearly 10.0 million cancer‐related deaths worldwide.^1^ Cancers can arise from various cell types and organs in the human body. They are characterized by uncontrolled cell proliferation, confirmed to be caused by random somatic genomic abnormalities. Through the accumulation of heritable genetic mutations, and the interactions with the surrounding microenvironment, as well as natural selection from cancer therapies, advantageous mutations can accumulate over time, while deleterious ones are eliminated.^2^ This evolutionary process enables cancer to develop phenotypes that promote survival and reproduction, leading to tumor progression, metastasis, and treatment resistance.^3^, ^4^

However, obtaining molecular information through invasive tissue sampling not only carries the risk of complications, posing dilemmas for pretreatment decision‐making and posttreatment patient monitoring, but is also quite time consuming. Meanwhile, the spatial heterogeneity within tumors causes a challenge for targeted therapies guided by regional sampling results. These therapies may only be effective against a subset of cancer cells, leaving other cancer subclones unaffected and potentially accelerating their growth, resulting in tumor evolution and recurrence. Besides, the high cost of advanced sequencing technology has limited its widespread use in clinical settings, making it particularly difficult for patients in medically disadvantaged areas to access easily.

Medical imaging represents a distinct and highly accessible method for acquiring tumor data compared with tissue sequencing. It allows for a macroscopic mapping of tumor cells, the microenvironment, and even the tissue surrounding the tumor at the voxel level, using noninvasive or minimally invasive multimodal imaging techniques. In recent years, computer technology has been integrated into medical imaging, enabling the high‐throughput extraction of quantitative features from medical images. Advanced machine learning (ML) and deep learning (DL) algorithms are then employed to analyze these features, facilitating a more effective assessment of large amounts of imaging data. In contrast to traditional imaging assessment methods, radiomic features offer a more objective and robust approach to capture tumor heterogeneity and reveal clinically significant higher‐order signatures that are not discernible to the human eye.^5^ Radiomics have been utilized to construct radiomic models, which have

Shown superior performance in noninvasive tumor stratification and prognosis assessment.^6^ Radiomics has emerged as a valuable addition to multiomics of cancer.

Radiogenomics, a new concept combines “radiomics” and “genomics,” has gained increasing attentions.^7^, ^8^ It involves the integration of advanced medical image analysis and multiomics data of tumors. Its goal is to uncover the relationship between radiomics and bio‐omics to pinpoint relevant biomarkers and build elaborate markers of disease and physiology and integrate multiple omics data for tumor diagnosis, classification, treatment decision, and prognosis.

In this review, we delineated the principal components of radiomics and genomics in oncology at the methodological level, elucidating their interconnections and integration mechanisms within the framework of radiogenomics with the aim of deconstructing the comprehensive landscape of radiogenomics. We also examine recent advancements in the application of radiogenomics to prevalent cancers such as glioma, lung cancer, and breast cancer, demonstrating its significant potential to address common clinical challenges. Finally, we highlight the current methodological challenges and limitations and discuss prospective directions for future research in the field.

## 2 RADIOMICS IN ONCOLOGY

**FIGURE 1:**
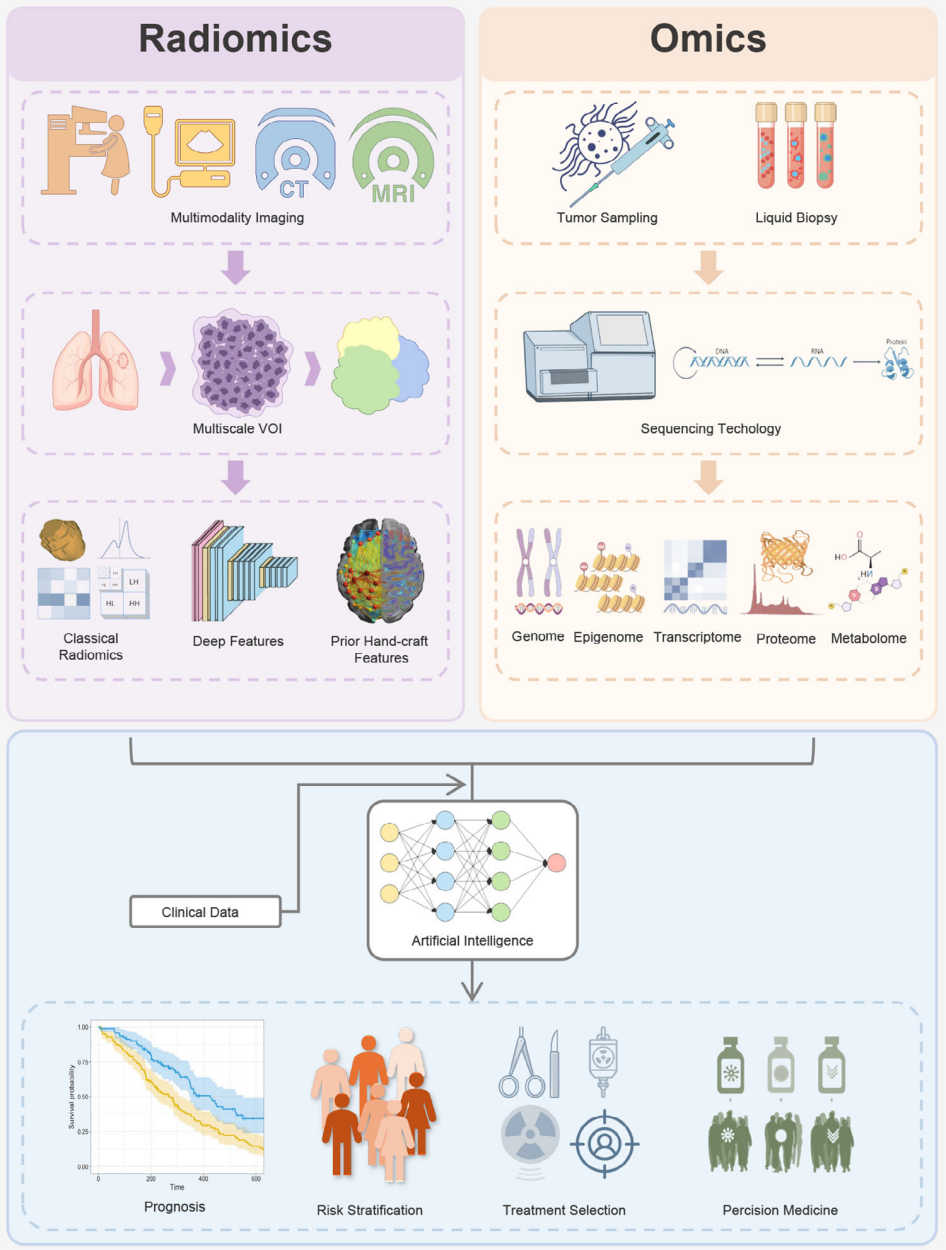
Schematic diagram illustrates the comprehensive integration of radiomics with omics data for precise cancer care. The first step involves collecting data resources, including imaging and biological samples. From these resources, various dimensions of radiomic features and molecular signatures of cancers are extracted and refined. Ultimately, radiomics and omics data are interconnected and integrated using advanced artificial intelligence algorithms to construct accurate clinical prediction models.

Radiomics is the high‐throughput mining of quantitative image features from standard‐of‐care medical imaging that enables data to be extracted and applied within clinical‐decision support systems to improve diagnostic, prognostic, and predictive accuracy.^5^, ^6^ In the following, we present three perspectives to illustrate the types of tumoral radiomic features obtained through various methods that can be utilized in a radiogenomics framework (Figure 1).

### 2.1 Features from multimodality images

Multimodality imaging technologies, such as digital radiography, computed tomography (CT), magnetic resonance imaging (MRI), nuclear medicine imaging such as positron emission tomography (PET), and others have been evolving for more than a century. These technologies employ different principles to capture various physical and chemical properties of tissues, offering a diverse range of imaging sequences. The radiomic features extracted from these multimodal images are often complementary, providing a multidimensional representation of tumors biology.

### 2.2 Features from multiscale of regions

Medical images acquired at different scales contain diverse biological information. Tumor‐level features, derived from both intratumor and peritumor regions, have been extensively utilized to characterize tumor heterogeneity. In addition, Subregion segmentation of cancers allows for multihabitat evaluation. However, it is important to note that cancer is not solely a localized disease; its occurrence, progression, and prognosis are often linked to the host organ or even the overall body condition. Radiogenomic studies have recently started incorporating the entire host organ, demonstrating predictive capabilities beyond tumor‐level profiling.^9^, ^10^ Anatomical multiscale radiomics enables a comprehensive assessment of cancer as a complex disease.

### 2.3 Approaches for feature extraction

#### 2.3.1 Classical radiomics

Classical radiomic features are widely utilized hand‐crafted features that are extracted from preprocessed images using predefined programs and specifications (such as pyradiomics) to describe radiographic aspects of shape, intensity, and texture.^11^ These features are derived from specific algorithms, which enhances their interpretability to some extent. Radiomic features are able to capture intratumor heterogeneity more effectively than the human eye, which is believed to explain the superiority of radiomics over traditional image analysis methods.

#### 2.3.2 Deep learning

DL techniques were used to automatically learn feature representations from medical images, eliminating the need for manual feature detection. DL methods offer several advantages such as reducing the need for preprocessing steps, enabling collaborative analysis of large volumes of high‐dimensional data, and providing superior problem‐solving capabilities. DL also allows for multitasking, including tumor segmentation, classification, and prognosis.^12^, ^13^, ^14^ Meanwhile, DL algorithms continue to evolve quickly and drive DL‐based radiomics forward.

#### 2.3.3 Priori hand‐crafted radiomics

In recent studies, new hand‐crafted radiomic features have been extracted batchwise as biomarkers for cancers, such as brain structure connectomics, tumor location, and the tumor field effect.^15^, ^16^, ^17^, ^18^ These features differ from classical ones in that they incorporate clinical prior knowledge, enabling them to capture specific pathophysiological information. This makes them advantageous for specific clinical tasks, particularly when dealing with limited amounts of data during model training.

## 3 GENOMICS IN ONCOLOGY

Generally, genomics is the study of all genes and DNA sequences of an organism. However, in the context of radiogenomics, the term “genomics” is often broadened to include the analysis of RNA, proteins, and other critical biomarker data that can reflect the origin and progression of tumor cells at the molecular level.^7^ Genomics should not be considered in isolation but rather in conjunction with transcriptomics, proteomics, metabolomics, and other “omics” disciplines.^19^, ^20^ Consequently, this review aligns with the prevailing academic perspective by integrating multiomics approaches within the framework of cancer radiogenomics. This integration is essential for radiogenomics to deliver a comprehensive biological and imaging‐based understanding of tumors.

### 3.1 Genomics from tumor tissue sampling

Obtaining samples directly from tumor tissues, including surgical resection, biopsy, and fine‐needle aspiration, is the most commonly used method of pathology sampling for genomics.^21^ In recent years, molecular pathology has played a pivotal role in facilitating precise diagnosis and informed treatment decisions for cancer, leveraging techniques such as immunohistochemical staining, in situ hybridization, and gene sequencing.^22^, ^23^ Furthermore, the integrated analysis of multiomics data based on high‐throughput sequencing platforms, gene chips, and mass spectrometry, enables a more comprehensive elucidation of cancer mechanisms and aids in the discovery of novel biomarkers for early cancer detection, prognosis assessment, and the identification of therapeutic targets.^19^, ^24^ Notably, the recently proposed spatial genomics technology holds the potential to unravel the intricacies of tumor heterogeneity, promising to localize and define tumor boundaries, subclones, and microenvironments at the molecular level.^25^

### 3.2 Genomics from liquid biopsy

Liquid biopsy has recently emerged as a promising sampling method for obtaining multiomics information from tumors. It primarily relies on blood samples to capture circulating tumor DNA, circulating tumor cells (CTCs), and exosomes present in the bloodstream, and detect the tumor‐derived multiomics biological information they carry. Liquid biopsy offers several advantages, including being noninvasive, highly reproducible, enabling early diagnosis, facilitating dynamic monitoring, and overcoming tumor heterogeneity.^26^, ^27^ Despite being in its infancy, liquid biopsy holds promise for promoting radiogenomics by furnishing highly time‐critical and continuously observable biomolecular data (Figure 1).

## 4 RADIOGENOMICS

### 4.1 Radiogenomics for precisive oncological molecular prediction

**FIGURE 2:**
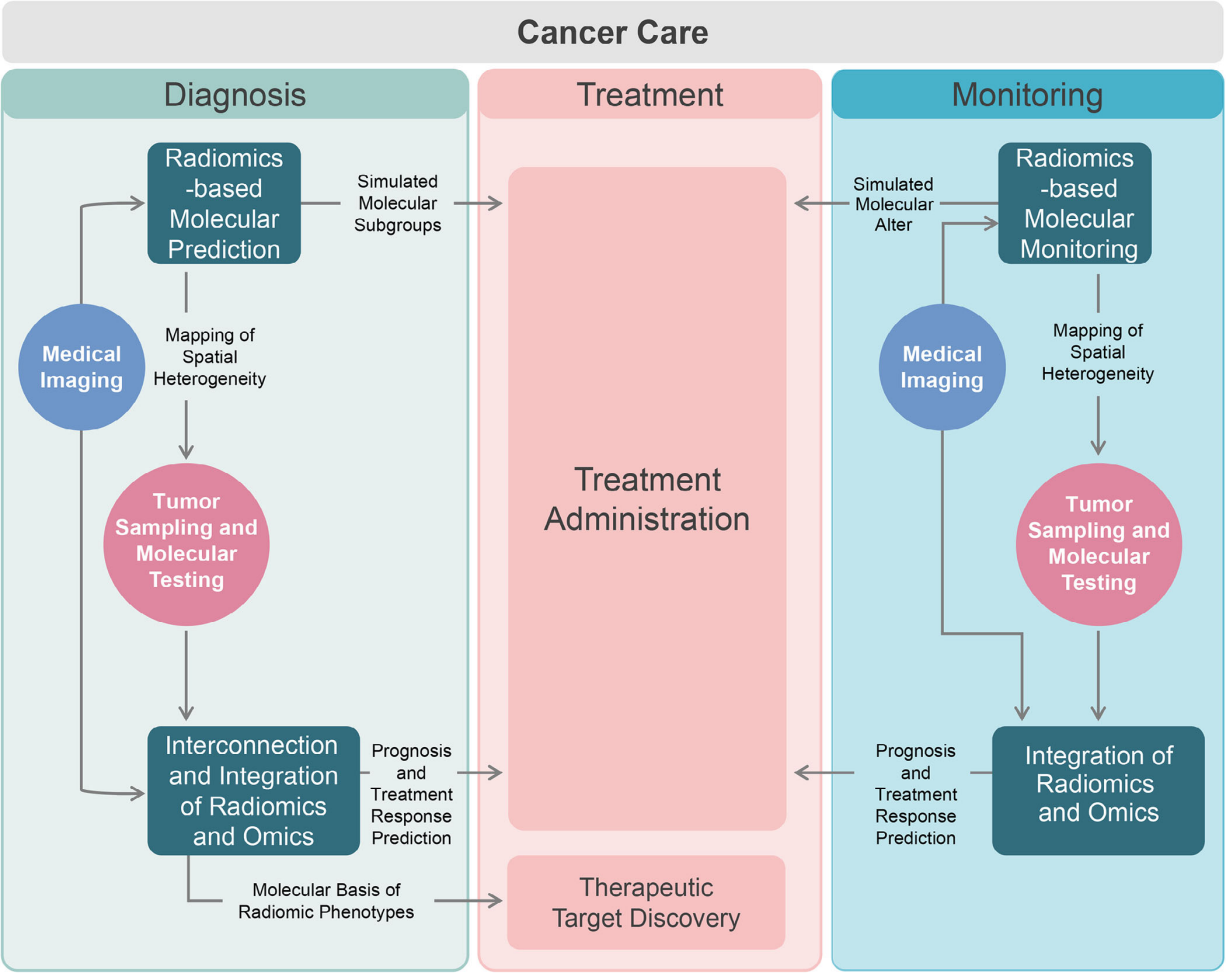
Potential enhanced clinical workflow with radiogenomics interventions. Radiogenomics offers the potential to noninvasively predict key molecular characteristics, including their temporal and spatial heterogeneity, at the initial diagnosis and posttreatment monitoring stages of cancer. This can help in discovering therapeutic targets, enhancing cancer prognosis, and predicting treatment response. Ultimately, radiogenomics can guide precision diagnosis and treatment of cancers, enhancing patient outcomes.

Investigating the association between radiomics and molecular biomarkers, with the aim of substituting invasive biomarkers with noninvasive and timely imaging markers for pre‐ and post‐treatment clinical decision‐making, plays a crucial role in radiogenomics (Figure 2).

#### 4.1.1 Prediction prior to treatment

Prior knowledge of tumor molecular subtypes before treatment is crucial for improved decision‐making in cancer care and is increasingly recognized as essential for neoadjuvant therapy (NAT).^28^, ^29^ Biopsy, the current gold standard, is inevitably invasive and prone to sampling errors due to tumor heterogeneity. Noninvasive Imaging, particularly with the emergence of radiomics, has demonstrated potential in predicting molecular subtypes throughout the entire tumor landscape, can provide a critical foundation for precise cancer treatment. In addition, by correlating with key biomolecules and pathways, biospecific radiomics features are likely to be more efficient and interpretable for determining tumor‐targeted treatment response and prognosis.

#### 4.1.2 Spatial heterogeneity landscaping

The spatial heterogeneity of tumor molecules within tumor subregions and among metastases has been extensively documented.^30^, ^31^ The accuracy of biopsy results has been questioned due to the limited amount of tissue sampled. While multipoint biopsies and Sequencing may address this limitation, they also increase risks of complications. Predicting the spatial distribution of key molecules in tumors through radiogenomics has potential to improve the reliability and representativeness of tissue sampling, reduce associated risks, and even guide precision radiotherapy.

#### 4.1.3 Molecular monitoring after treatment

Adaptive changes in the tumor genome from anticancer therapy are a key driver of treatment resistance.^32^ Real‐time monitoring of these changes could underpin timely therapy adjustment and new targeted development. However, biopsy‐based molecular monitoring is often delayed and risky for repeated tests. Radiogenomic monitoring of tumor genomes noninvasively shows promise for precision medicine's timely implementation based on dynamic genomic shifts.

### 4.2 Radiogenomics for risk stratification and prognosis evaluation

Numbers of key biomarkers for tumor treatment and prognosis have been identified and partially implemented into clinical practice. However, even with the latest findings it is difficult to predict tumors perfectly. Radiomics’ universality, noninvasiveness, and ability to observe biological information at the tumor and even organ level make it a powerful complement to current molecular typing systems for cancer, which is expected to further facilitate precise and individualized treatment (Figure 2).

#### 4.2.1 Prognosis stratification

Cancer outcomes, such as remission, progression, complications, death, and altered quality of life, are of paramount concern. Accurate prognosis stratification help to inform patients about the future course of disease and to guide doctors and patients in joint decisions on further treatment, as well as to facilitate of clinical research to develop new treatment options. The integration of multidimensional biological information, such as radiogenomics, promises a more comprehensive path to accurate prognosis.

#### 4.2.2 Prediction of response to therapy

Advances in radiochemotherapy, targeted therapy, and immunotherapy has provided additional options for tumor treatment. These treatments elicit diverse responses within the patient population and are accompanied by varying degrees of side effects. Tumor genomics and other invasive biomarkers are utilized to stratify patients and select appropriate candidates for therapy. However, the limitations of regional biopsies hinder the accurate prediction of treatment benefit based on molecular information. Radiogenomics is expected to leverage the respective strengths of molecular markers and imaging to facilitate precision oncology decision making.

## 5 APPLICATION OF RADIOGENOMICS TO CANCER CARE

To provide a comprehensive overview of the advancements in radiogenomics across multiple facets of cancer care, this review focuses on the recent evidence from the past 5 years in three highly researched cancer types: glioma, lung cancer, and breast cancer (Tables 1, 2, 3). We also briefly outlined the recent evidence of radiogenomics in other cancers (Table 4). Additionally, we discuss the current challenges and future directions for further investigation.

**TABLE 1:** Summary of recent key studies on radiogenomics decoding of gliomas.

Subset	Application	Molecular data	Modality	Radiomic features	Training and validation cohorts	Public data sources	Results	References
Brainstem gliomas	Prediction of molecular	H3k27M	MRI (conventional, diffusion)	Classical radiomics, connectomics	Training, *n* = 93 Temporal validation, *n* = 40	No	AUC of 95.31% from combined model, outperformed radiomics and connectomics alone	^16^
IDH‐mutant astrocytoma	Prediction of molecular	CDKN2A/B	MRI (conventional)	Deep learning	Training, *n* = 234 Cross‐validation	TCGA‐TCIA	Average AUC of 97.04% from deep learning model	^33^
Glioma	Prediction of molecular	IDH	MRI (conventional)	Classical radiomics, location features	Training, *n* = 679 Intersite cross‐validation	TCGA‐TCIA	Maximum AUC of 79.1% from the combined model, outperformed radiomic model	^18^
Intramedullary gliomas	Prediction of molecular	ATRX, P53	MRI (conventional)	Classical radiomics	Training, *n* = 229 External validation, *n* = 129	No	AUCs of 0.7622 and 0.7954 in predicting ATRX and P53, respectively	^34^
Glioma	Prediction of molecular	Transcriptome	MRI (conventional)	Classical radiomics	Training, *n* = 130 External validation, *n* = 55	CGGA	AUC of 0.924 for identifying immune subtypes	^35^
IDH‐mutant low‐grade glioma	Prediction of molecular	ATRX	MRI (conventional, perfusion, diffusion), 18F‐FDG PET	Classical radiomics	Training, *n* = 72 External validation, *n* = 30	No	AUC of 0.975 from PET+ADC+CE‐T1WI model	^36^
Glioma	Prediction of molecular	IDH, 1p19q	MRI (conventional)	Deep learning	Training, *n* = 1508 External validation, *n* = 240	TCGA‐TCIA, BraTS	AUCs of 0.90 and 0.85 for the IDH and 1p/19q prediction	^12^
Glioma	Prediction of molecular	IDH	MRI (conventional, brain network)	Deep learning	Training, *n* = 270 Internal validation, *n* = 117	TCGA‐TCIA	AUC of 96.2%, outperformed published baseline	^15^
Glioma	Prediction of molecular	IDH and MGMT	MRI (conventional)	Classical radiomics	Training, *n* = 159 External validation, *n* = 189	TCGA‐TCIA	AUC of 0.866	^37^
Glioma	Prediction of molecular	MGMT	MRI (conventional)	Deep learning	N up to 985	BraTS	Most (80.2 and 60.0%) of the 420 developed models showed negative results in terms of test accuracy and test AUC	^38^
Glioma	Prediction of molecular	IDH, 1p19q	MRI (conventional, diffusion)	Deep learning	Training, *n* = 384 External validation, *n* = 147	TCGA‐TCIA	Correctly classifying 95.2, 88.9, 60.0% of the three subtypes; better performance achieved using 3‐class structure and diffusion MRI	^39^
Midline pediatric high‐grade glioma	Prediction of molecular	H3k27M	MRI (conventional, diffusion)	Classical radiomics	Training, *n* = 76 Internal validation, *n* = 31	No	AUC of 0.92	^40^
Pediatric low‐grade glioma	Prediction of molecular	Braf	MRI (diffusion)	Classical radiomics	Training, *n* = 299 External validation, *n* = 23	No	Average AUC of 0.74	^41^
Glioma	Prediction of molecular	IDH, 1p19q	MRI (conventional)	Classical radiomics, deep learning	Training, *n* = 780 Internal validation, *n* = 236	No	Maximum AUC of 4 tasks range from 0.68 to 0.89; deep learning outperformed radiomics in most tasks	^42^
Brainstem gliomas	Prediction of molecular	H3k27M	MRI (APTw)	Classical radiomics	Training, *n* = 64 Temporal validation, *n* = 29	No	Accuracy of 0.86	^43^
IDH‐wildtype glioma	Prediction of molecular	TERT	18F‐FET PET	Classical radiomics	Training, *n* = 112 Internal validation, *n* = 47	No	AUC of 0.61	^44^
Glioblastoma	Radiogenomic correlation	Transcriptome	MRI (conventional)	Classical radiomics	Training, *n* = 125 External validation, *n* = 22	TCIA	Difference found in radiomic phenotypes and signaling pathways between sexes	^45^
Glioma	Prediction of molecular	IDH, 1p19q, TERT	MRI (conventional, diffusion)	Classical radiomics	Training, *n* = 238 Internal validation, *n* = 119	No	AUCs of 0.884, 0.815, and 0.669 for predicting IDH, 1p19q, and TERT status; similar prognosis shown with actual subtypes	^46^
Glioblastoma	Prediction of molecular	MGMT	18F‐DOPA PET	Classical radiomics	Training, *n* = 59 Internal validation, *n* = 10	No	AUC of 0.80	^47^
Glioblastoma	Prediction of molecular	Genome	MRI (conventional, perfusion, diffusion)	Classical radiomics	Training, *n* = 85 Internal validation, *n* = 35	No	AUCs of 0.88, 0.76, and 0.81 for the prediction of RTK, P53, and Rb pathways	^48^
Low‐grade glioma	Prediction of molecular	Genome	MRI (conventional)	Deep learning	Training, *n* = 182 Cross‐validation	TCGA‐TCIA	AUC of 0.698 for cluster coc1 vs. coc2 subtypes, 0.731 for cluster coc2 vs. coc3	^49^
Glioma	Prediction of molecular	IDH	MRI (conventional, perfusion)	Deep learning	Training, *n* = 395 Internal validation, *n* = 18	No	AUC of 0.95	^50^
Glioblastoma	Prediction of molecular	POSTN	MRI (conventional)	Classical radiomics	Training, *n* = 93 (patients)/40 (OXs) Cross‐validation	No	AUC of 76.56% in patients and 92.26% in OXs; radiomic features in OXs were significantly associated with those in patients	^51^
Glioblastoma	Radiogenomic correlation	Genome and transcriptome	MRI (conventional)	Deep learning	Training, *n* = 127 External validation, *n* = 389	TCGA‐TCIA, CGGA	Deep learning signature correlate with RTK, P53 and RB pathways, and CDKN2A deletion	^52^
Glioma	Prognosis: OS	IDH	MRI (conventional) of whole brain	Deep learning	Training, *n* = 935 External validation, *n* = 465	TCIA	AUCs ranged between 0.77 and 0.94, outperformed model that require ROI	^9^
Glioma	Prognosis: OS; Radiogenomic correlation	Genome and transcriptome	MRI (diffusion)	Deep learning	Training, *n* = 688/78 External validation, *n* = 1320	TCGA‐TCIA, CGGA	Deep learning signature improved prognosis of clinic‐molecular model, and correlated with five pathways	^53^
Low‐grade glioma	Prognosis: OS; Benefit from chemotherapy	IDH	MRI (conventional)	Classical radiomics	Training, *n* = 149 External validation, *n* = 66	No	Radiomics joint with clinicopathologic data outperformed the clinicopathologic data alone (C‐index, 0.821 vs. 0.692)	^54^
Glioblastoma	Prognosis: OS	Genome	MRI (conventional, perfusion, diffusion)	Classical radiomics	Training, *n* = 571 Cross‐validation	No	Radiogenomics subtype for risk stratification at a hazard ratio of 1.64	^55^
Glioma	Prognosis: OS	Multiomics	MRI (conventional)	Classical radiomics	Training, *n* = 111 External validation, *n* = 53	TCGA‐TCIA, GEO, cbioportal, CCLE, GDSC, GO	Radiomics subtype for risk stratification at a hazard ratio of 2.70	^56^

Abbreviations: 18F‐DOPA, 18F‐dihydroxyphenylalanine; 18F‐FDG, 18F‐fluoro‐d‐glucose; 18F‐FET, 18F‐fluoro‐ethyl‐tyrosine; APTw, amide proton transfer‐weighted; AUPRC, area under the precision‐recall curve; BraTS, The Brain Tumor Segmentation challenge; CCLE, cancer cell line encyclopedia; CGGA, Chinese glioma genome atlas; GDSC, Genomics of drug sensitivity in cancer; GEO, the gene expression Omnibus; GO, the gene ontology; OS, Overall Survival; OXs, orthotopic xenografts; TCGA, the Cancer Genome Atlas Program; TCIA, the Cancer Image Archivexvv.

**TABLE 2:** Summary of recent key studies on radiogenomics decoding of lung cancers.

Subset	Application	Molecular data	Modality	Radiomic features	Training and validation cohorts	Public data sources	Results	References
NSCLC	Prediction of molecular	PD‐L1	CT	Classical radiomics	Training, *n* = 62 External validation, *n* = 109	TCGA‐TCIA	AUCs of 0.70, 0.72. and 0.66 at expression >1, >5, and >90%; potential prediction of response to PD‐1 or PD‐L1 treatment, pneumonia development, and patient survival	^57^
NSCLC	Radiogenomic correlation; Prognosis: distant recurrence	Genome associate with distant recurrence	18F‐FDG PET	Classical radiomics	Training, *n* = 34 Internal validation, *n* = 19	TCGA‐TCIA	AUC of 0.912 from combine model	^58^
NSCLC	Prediction of molecular	EGFR, ALK, ERBB2, BRAF, MET, ROS1, RET, KRAS, TP53, and PD‐L1	CT	Classical radiomics, deep learning	Training, *n* = 877 Internal validation, *n* = 110	No	AUCs of 0.856 to 0.877 for 4 tasks from radiomics and deep learning combined models	^59^
Lung cancer	Prediction of molecular; Prognosis: after EGFR‐TKI treatment	EGFR	CT (whole‐lung)	Deep learning	Training, *n* = 10,427 External validation, *n* = 8375	TCIA	AUCs of 0.748–0.813; outperformed models based on ROI; helped identify TKI resistance; associated with genotype and pathways linked to drug resistance and progression	^10^
NSCLC	Prediction of molecular	EGFR, KRAS	CT, 18F‐FDG PET	Classical radiomics	Training, *n* = 94 Internal validation, *n* = 42	TCIA	AUCs of 0.92 to 0.94 for EGFR and 0.91 to 0.94 for KRAS; combat harmonization improve performance	^60^
NSCLC	Prediction of molecular	PD‐L1	CT	Classical radiomics, deep learning	Training, *n* = 908 Internal validation, *n* = 227	No	AUCs of 0.950, 0.934, and 0.946 for predicting PD‐L1 expression signature <1, 1−49, and ≥50%; improved prognosis of clinical data	^61^
NSCLC	Prediction of molecular	EGFR status and subtypes, PD‐L1	CT (tumor and whole lung)	Classical radiomics, deep learning	Training, *n* = 3053 Internal validation, *n* = 763	No	AUCs of 0.841 to 0.905 for 5 predicting tasks from joint module	^62^
LUAD	Prediction of molecular	T790M mutation	CT	Classical radiomics	Training, *n* = 186 Internal validation, *n* = 74	No	AUCs of 0.71 and 0.76 from radiomics, and nomogram models;	^63^
NSCLC	Radiogenomic correlation	Genome	CT	Deep learning	Training, *n* = 142 External validation, *n* = 71	No	Deep learning score was associated with pathways and antitumor immune cell infiltration in the microenvironment	^64^
LUAD	Radiogenomic correlation	Genomic alterations	CT	Classical radiomics	*n* = 219	No	Associations found between radiomic subset and clinical‐pathologic, genomic features, and outcomes	^65^
NSCLC	Prediction of molecular	ALK fusion	CT	Deep learning	Training, *n* = 651 External validation, *n* = 286	No	AUCs of 0.775 and 0.848 from CT and CT‐clinicopathological combined model; stratified prognosis under ALK‐TKI treatment	^66^
NSCLC	Prediction of molecular	PD‐L1	CT	Deep learning	Training, *n* = 750 Internal validation, *n* = 96	No	AUC of 0.76, stratified prognosis under anti‐PD‐1 antibody treatment	^67^
NSCLC	Prediction of molecular	EGFR, PD‐L1	18F‐FDG PET/CT	Deep learning	Training, *n* = 429 External validation, *n* = 65	No	AUCs of 0.81 and 0.84 from deep learning and combined model; stratified prognosis under TKIs and ICIs treatment	^68^
NSCLC	Prediction of molecular	TMB	CT	Deep learning	Training, *n* = 236 Internal validation, *n* = 65	No	AUC = 0.81; stratified prognosis under ICIs treatment	^69^
LUAD	Prediction of molecular	Cytact	18F‐FDG PET	Deep learning	Training, *n* = 93 External validation, *n* = 59	TCGA‐TCIA	Predicted cytact positively correlated with ground truth; stratified prognosis under ICB treatment	^70^
NSCLC	Response to ICIs	Plasma extracellular vesicle PD‐L1	CT	Classical radiomics	Training, *n* = 27 Internal validation, *n* = 30	No	Radiogenomics model increase specificity, sensitivity, and accuracy of ICIs response prediction compared with genomics	^157^
NSCLC	Prediction of metastasis	Genome	18F‐FDG PET	Deep learning	Training, *n* = 93 Cross‐validation	No	Highest AUC of 0.855, outperformed radiomic and genomic models	^71^

Abbreviations: ALK, Anaplastic Lymphoma Kinase; cytact, cytolytic activity score; ICB, immune checkpoint blockade; ICIs, Immune Checkpoint Inhibitors; KRAS, Kirsten Rat Sarcoma Viral Oncogene Homologue; LUAD, lung adenocarcinoma; PD‐1, Programmed Death 1; PD‐L1, Programmed Cell Death Ligand 1; MET, Mesenchymal Epithelial Transition; ROS1, ROS proto‐oncogene 1; RET, Rearranged During Transfection; TKI, Tyrosine Kinase Inhibitors; TMB, Tumor Mutation Burden.

**TABLE 3:** Summary of recent key studies on radiogenomics decoding of breast cancers.

Subset	Application	Molecular data	Modality	Radiomic features	Training and validation cohorts	Public data sources	Results	References
Breast cancer	Prediction of molecular	HER2	MRI (conventional)	Deep learning	Training, *n* = 329 External validation, *n* = 61	No	AUCs of 0.76 and 0.75 for prediction of HER2‐overexpressing and HER2‐low‐positive; stratified prognosis	^72^
Breast cancer	Prediction of molecular change after NAT	ER‐/HER2‐ or ER‐low/HER2‐	MRI (conventional, perfusion, diffusion)	Classical radiomics	Training, *n* = 66 Internal validation, *n* = 19	No	AUC of 0.86	^73^
Breast cancer	Prediction of molecular	TNBC and transcriptomic TNBC subtypes	MRI (conventional)	Classical radiomics	Training, *n* = 420 External validation, *n* = 164	No	AUCs of 0.613 to 0.723 for identification of TNBC; AUCs of 0.598–0.796 for distinguishing TNBC subtypes; peritumoral radiomic features were associated with immune suppression and upregulated fatty acid synthesis	^74^
Breast cancer	Prediction of molecular	HR, HER2, Ki‐67	MRI (conventional, perfusion)	Classical radiomics	Training, *n* = 218 Internal validation, *n* = 73	No	Maximum AUC of 0.8 for prediction of molecular from texture features using random forest in SSF of 0	^75^
Breast cancer	Prediction of molecular	ER, PR, PAM50	MRI (conventional, perfusion)	Deep learning	Training, *n* = 585 Cross‐validation	No	AUCs of 0.942 and 0.920 for predicting ER and PR, 0.742 for PAM50; performance improved when peritumor region included	^76^
Breast cancer	Prediction of molecular	HR, HER2	Contrast‐enhanced spectral mammography	Classical radiomics	Training, *n* = 164 Internal validation, *n* = 18	No	ACCs of 0.89 and 0.85 for predicting HER2 and HR	^77^
Breast cancer	Prediction of molecular	CD8+ T cells‐based immunophenotype	MRI (conventional)	Classical radiomics	Training, *n* = 137 Internal validation, *n* = 45	No	AUCs of 0.985 and 0.984; associated with complete response to NAC	^78^
Breast cancer	Prediction of molecular	Ki‐67, luminal A subtype	MRI (conventional, perfusion)	Deep learning	Training, *n* = 122 Internal validation, *n* = 80	No	AUCs of 0.819 and 0.799 for predicting Ki‐67 and luminal A	^79^
TNBC	Prediction of molecular	Transcriptome	MRI (conventional)	Classical radiomics	Training, *n* = 98 Internal validation, *n* = 41	No	AUC of 0.79, related to activated immune‐related pathways and hot immune microenvironment	^80^
Breast cancer	Prediction of molecular	Immunohistochemistry subtype	Ultrasound	Deep learning	Training, *n* = 1275 External validation, *n* = 845	No	MCCs of 0.59–0.79 for predicting 4 subtypes; MCCs of 0.54 and 0.65 for discriminate luminal and nonluminal	^81^
Breast cancer	Prediction of molecular	Transcriptome	MRI (conventional)	Classical radiomics	Training, *n* = 96 External validation, *n* = 155	TCGA‐TCIA	AUC of 0.815 for predicting immunoscore, radiomics signature associated with recurrence‐free and overall survival rates	^82^
Breast cancer	Prediction of molecular	PD‐L1	MRI (conventional)	Classical radiomics	Training, *n* = 62 Cross‐validation	No	AUC of 0.904	^83^
Breast cancer	Prediction of molecular	Tumor microenvironment subtype	MRI (perfusion)	Deep learning	Training, *n* = 342 Cross‐validation	TCGA‐TCIA	Radiomics features are correlated with markers of breast TME, such as ER, PR, HER2, PD‐1, PD‐L1, EGFR	^84^
Breast cancer	Prediction of molecular	Immunohistochemistry subtype	MRI (conventional, perfusion)	Classical radiomics	Training, *n* = 211 Cross‐validation	No	Maximum AUC of 0.832 from radiomic model based on tumor subregion related to fast‐flow kinetics, AUC increased to 0.897 in the tumor‐ and parenchyma‐based predictive modal	^85^
Breast cancer	Prediction of molecular	HER2	Ultrasound (video)	Deep learning	Training, *n* = 357 Internal validation, *n* = 88	No	AUCs of 0.72 and 0.81 from radiomic and combine model	^86^
Breast cancer	Prediction of molecular	Immunohistochemistry subtype	Mammography, ultrasound	Deep learning	Training, *n* = 2688 Internal validation, *n* = 672	No	MCC of 0.837 for predicting 4‐category subtypes; AUC of 0.929 for discriminate luminal and nonluminal	^87^
Breast cancer	Prediction of molecular	HR	Mammography	Deep learning	Training, *n* = 2083 Temporal validation, *n* = 190	No	Average AUC of 0.92	^88^
Breast cancer	Response to NAC: pCR; prognosis: OS, RFS	IL‐17 and estrogen signaling pathways	MRI (perfusion)	Classical radiomics	Training, *n* = 255 External validation, *n* = 174	TCGA‐TCIA	Radiomics predicted tumor shrinkage with an AUC of 0.886 and PCR with an AUC of 0.760, correlating with IL‐17 and estrogen signaling pathways	^89^
Breast cancer	Prognosis: PFS	Immunohistochemistry subtype, PD‐L1, Ki67	CT	Classical radiomics	Training, *n* = 171 Internal validation, *n* = 69	No	AUC of 0.961 for prognosis under ICIs‐based therapies from the integrated clinical‐radiomics model	^90^
TNBC	Response to NAC: pCR	Genome: the variant allele frequency features	MRI (pre‐ and posttreatment)	Classical radiomics	Training, *n* = 75 Internal validation, *n* = 37	No	AUCs of 0.87 from radiogenomic model, outperformed radiomic model; two highly frequent mutations related to epirubicin resistance	^91^
TNBC	Response to NAC, Prognosis: DFS	511 genes related to the development and targeted therapy	MRI (conventional, perfusion)	Classical radiomics	Training, *n* = 413 Internal validation, *n* = 77	TCGA‐TCIA	AUC of 0.93 for predicting PCR from radiogenomic models; significantly stratify patients by disease‐free survival	^92^
Breast cancer	Predict lymph node metastasis and therapeutic response	Whole‐transcriptome	MRI (conventional)	Classical radiomics	Training, *n* = 103 External validation, *n* = 924	TCGA‐TCIA	Radiogenomics nomogram identified axillary lymph node metastasis and drug therapeutic response at a statistically significant level (*p* < 0.05)	^93^

Abbreviations: DFS, disease‐free survival; HR, Hormone Receptor; IL‐17, the Interleukin‐17; MCC, Matthews correlation coefficient; NAC, Neoadjuvant Chemotheropy; NAT, Neoadjuvant Theropy; pCR, pathologic Complete response; RFS, Relapse‐free Survival.

**TABLE 4:** Summary of key studies on radiogenomics decoding of colorectal cancer, renal cell carcinoma and prostate cancer.

Subset	Application	Molecular data	Modality	Radiomic features	Training and validation cohorts	Public data sources	Results	Reference
Colorectal cancer	Prediction of molecular	RAS	CT	Deep learning	Training, *n* = 208 Internal validation, *n* = 23	No	AUC of 0.955	^94^
Colorectal cancer	Prediction of molecular	DNA mismatch repair status	CT	Deep learning	Training, *n* = 1124 External validation, *n* = 206	No	AUC of 0.915; similar satisfying prediction performance showed in subgroup analysis	^95^
Colorectal cancer (stage IV)	Prediction of molecular	TMB	18F‐FDG PET	Classical radiomics	Training, *n* = 91 Cross‐validation	No	AUC of 0.719	^96^
Colorectal cancers with liver metastasis	Prediction of molecular	CD73 expression	CT	Classical radiomics; Deep learning (liver metastasis)	Training, *n* = 125 Internal validation, *n* = 35	No	AUC of 0.79 from deep learning model; outperformed other models; prognostic value of radiogenomics was independent of the standard clinical risk score	^97^
Colorectal cancers with liver metastasis	Prediction of molecular	RAS and BRAF mutation	CT	Classical radiomics (liver metastasis)	Training, *n* = 124 Internal validation, *n* = 35	No	AUC of 0.79	^98^
Colorectal cancer	Prognosis: DFS	Four genomic subclones identified unsupervisedly	CT	Classical radiomics	Training, *n* = 236 External validation, *n* = 69	Gene Expression Omnibus	Radiogenomic signatures were independent prognosis factor; associated with extracellular matrix and immune‐related pathways	^99^
ccRCC	Prediction of molecular	Immune‐related genomic signature	CT	Classical radiomics	Training, *n* = 135 Internal validation, *n* = 58	TCGA‐TCIA	AUC of 0.72 for predicting immune‐related molecular subtypes	^100^
ccRCC	Prediction of molecular; Prognosis: OS	Transcriptome	CT	Classical radiomics	Training, *n* = 127 External validation, *n* = 75	TCGA‐TCIA	Lipid metabolic pathway‐specific radiogenomics modeling is an independent risk factor for patient prognosis	^101^
ccRCC	Prediction of molecular; Prognosis: OS	Genome, transcriptome, proteome	CT	Classical radiomics	Training, *n* = 104 Internal validation, *n* = 103	TCGA‐TCIA	AUCs of 0.949–0.973 for predicting 4 genetic mutations and 4 mRNA‐based subtypes; highest AUC achieved for prognosis using radiogenomic models	^102^
Prostate cancer	Radiogenomic correlation	CTCs count and plasma cfDNA level	CT	Classical radiomics (bone metastasis)	*N* = 8	No	Radiomic features consistently and strongly positively correlated with CTCs count, plasma CFDNA CTCs clusters 6, 7, and 8	^103^
Prostate cancer	Radiogenomic correlation	Genotypes for apoptosis, hypoxia, and androgen receptor expression	MRI (conventional, diffusion)	Classical radiomics	*N* = 8	No	Significant correlation observed between radiomic features and CNV of genes associated with apoptosis, hypoxia, and androgen receptor (*p* ≤ 0.05)	^104^

Abbreviations: ccRCC, Clear Cell Renal Cell Carcinoma; cfDNA, cell‐free DNA; CNV, copy number variation; CTCs, Circulating tumor Cells.

### 5.1 Glioma

Gliomas are heterogeneous entities, which characterized by specific gene alter.^105^ Although some gliomas are benign and have a favorable prognosis, the majority, particularly glioblastoma, are highly fatal.^106^ This is not only due to the direct impact of the tumor on the structure and function of the brain, but also because of the risk of serious complications during invasive procedures against the lesion.^107^, ^108^, ^109^ Refining medical decisions with precision for optimal outcomes at minimal cost is vital. Medical imaging plays a pivotal role in assessing gliomas noninvasively. Radiogenomics holds significant potential in predicting the molecular subtypes of gliomas preoperatively and stratifying patients’ prognosis.

Isocitrate dehydrogenase (IDH) mutation and 1p19q chromosome codeletion serve as the key determinants for the classification of adult diffuse glioma.^105^ These genetic alterations not only indicate distinct prognoses but also guide diverse treatment strategies. In several large‐scale studies,^12^, ^13^, ^18^, ^42^ radiomic models have shown the ability to predict IDH or 1p19q status either independently or within a comprehensive framework for adult diffuse glioma. DL models often exhibit superior performance than classical radiomics, even when applied to the same dataset. In particular, Van et al.’s DL model achieved high accuracy in externally validating molecular predictions, while also in performing multitask of tumor grading and segmentation. This highlights the significant advantages of high‐performance and multitasking capabilities in DL, especially when trained with ample data. Utilizing a single model to predict subtypes of IDH and 1p19q offers greater accessibility compared with predicting them individually. Cluceru et al.^39^ concluded that a three‐class model for subtyping has superior generalization capability compared with a two‐tiered approach. This may be attributed to the significant reduction in training cases during the second step of the two‐tiered pattern, whereas the three‐class approach incorporates all data for model training.

Conventional MRI is superior in displaying anatomical structures, while other advanced imaging techniques, such as diffusion‐weighted imaging and perfusion‐weighted imaging, have been developed to display or amplify microenvironmental information, providing multidimensional data for molecular prediction of tumors. Radiogenomic studies have shown improved accuracy and stability of the model, although further external validation data are still required.^39^, ^46^, ^50^, ^110^, ^111^ In addition to tumor signal, features from tumor location, which have been shown to correlate with IDH status and are less influenced by image acquisition and measurement variability, as well as brain network connectome,^112^ which can identify disrupted white matter tracts and reveal hidden tumor invasion, have also been incorporated into radiogenomic models to enhance accuracy and generalization in predicting IDH status.^15^, ^18^

Substitution of lysine 27 to methionine in histone H3 (H3K27M) characterizes a subset of highly malignant pediatric gliomas that are unresectable and exhibit rapid progression with a dismal prognosis.^105^ This has been proven to be a significant prognostic factor for overall survival, irrespective of age, tumor location, or histopathological grading in midline gliomas.^113^, ^114^ Radiomic models utilizing Conventional MRI have achieved area under the receiver operating characteristixc curves (AUCs) ranging from 0.78 to 0.85 in identifying H3K27M in midline glioma.^115^, ^116^ Moreover, the integration of brain structural connectomics or diffusion‐weighted imaging has shown the ability to further enhance the precision of the models.^16^, ^40^ Zhou et al.^43^ have recently demonstrated the efficacy of radiomic models based on amide proton transfer weighted MRI, an emerging functional imaging technique, in predicting H3K27M in pontine gliomas, with an accuracy of 0.86 in an independent prospective cohort.^43^ Although current radiogenomic predictions for H3K27M still lack multicenter external validation, these findings hold great promise, particularly with the application of multimodal imaging techniques.

O6‐methylguanine‐DNA‐methyltransferase (MGMT) promoter methylation serves as a significant molecular marker for assessing the therapeutic efficacy of alkylating agents like temozolomide, which is a first‐line chemotherapy drug for glioma.^117^ Despite numerous attempts to construct radiogenomic models for the prediction of MGMT status, either the results were far from satisfactory or lacked adequate external validation.^118^ The most recent systematic review indicates substantial heterogeneity in the results of MRI radiomics models for predicting the methylation status of MGMT in grade IV gliomas, with low performance observed in external validation.^119^ Two large‐scale external validations of previous research findings published in 2022 and 2023 also demonstrate that these MRI‐based radiomics models are still insufficient in accurately predicting MGMT in gliomas prior to surgery.^38^, ^120^ A radiogenomic model based on PET indicated higher accuracy (AUC = 0.80 in cross‐validation) in predicting MGMT status, but further validation was required.^47^ Nonetheless, it is worth exploring the use of multimodal images based radiomics to enhance prediction accuracy. Interestingly, the co‐occurrence of IDH mutation and MGMT methylation characterizes a subtype of gliomas with a favorable prognosis and potential benefits from temozolomide, and this can potentially be predicted using radiomic models.^37^

Other molecular markers, such as alpha‐thalassemia mental retardation X‐linked (ATRX), telomerase reverse transcriptase (TERT), EGFR, tumor protein 53(TP53), cyclin‐dependent kinase inhibitor 2A/B (CDKN2A/B), proto‐oncogene B‐Raf and v‐Raf murine sarcoma viral oncogene homlog B (BRAF), cyclin D1 (CCND1), and cyclin‐dependent kinases 6 (CDK6), have also emerged as crucial factors in glioma classification, prognosis, and targeted therapy^121^, ^122^ and have become focal points for radiogenomic investigations.^33^, ^34^, ^36^, ^41^, ^44^, ^46^, ^123^, ^124^, ^125^, ^126^, ^127^ However, currently, there is insufficient evidence to support the clinical application of radiogenomic models for these markers. Notably, Zinn et al.^51^ developed a radiomics model to predict the expression level of periostin in glioblastoma, and importantly, they confirmed the causal relationship between radiomics subtypes and molecular expression through simultaneous radiomics analysis on orthotopic xenografts. As new molecular markers for gliomas are gradually integrated into clinical practice, further efforts are needed to establish substantial evidence regarding the application of radiogenomics to these relatively rare markers.

Thanks to microarray and next‐generation sequencing technologies, oncology research has made significant strides in comprehensively analyzing the molecular landscape of cancer cells and the tumor microenvironment, which goes beyond merely detecting specific genetic alterations. Several radiogenomic studies have revealed the intense associations between radiomic phenotypes and multiomics molecular subtypes and the tumor immune microenvironment (TIME).^71^, ^72^, ^73^ Hu et al.^128^ attempted to correlate radiomics with the genetic status of various subregions of the tumor. They collected 48 image‐guided biopsies from 13 glioblastomas and confirmed the spatial heterogeneity of genetic subtypes within the tumor, which correlated with radiomic features. Several studies have found correlations between MR radiomics prognostic phenotypes and specific molecular signaling pathways and intercellular communication in gliomas.^48^, ^52^, ^53^, ^129^ Recent studies have defined new phenotypic subtypes of gliomas based on radiomics or radiogenomics and found significant differences in survival, immune infiltration, and drug susceptibility among these subtypes, providing a better understanding of the molecular basis of phenotypic characterization of gliomas.^55^, ^130^ These findings reveal the underlying biological mechanisms behind radiomic models and may be used to identify potential therapeutic targets for gliomas.^53^ In particular, Beig et al.^45^ investigated the radiogenomic associations of MRI‐based phenotypes with transcriptomic data in male and female patients. Their aim was to identify the signaling pathways that drive sex‐specific tumor biology and treatment response in glioblastoma.

Radiomics is believed to capture tumor heterogeneity and provide additional biological information beyond the tumor, making it a valuable complement to molecular biomarkers used in clinical practice.^131^, ^132^ Whether in low‐grade gliomas, high‐grade gliomas, or overall diffuse gliomas, radiogenomic models showed superior performance in stratifying patient prognosis compared with classical radiomic or molecular‐clinical models.^9^, ^52^, ^133^ By integrating genetic data such as IDH and MGMT status with radiomics, radiogenomic models can more accurately differentiate postoperative recurrence from pseudoprogression and assess the efficacy of chemotherapy.^54^, ^134^

### 5.2 Lung cancer

Lung cancer is one of the most frequently diagnosed cancers and the leading cause of cancer‐related deaths worldwide.^135^ While surgical resection remains the preferred treatment modality, advancements in chemoradiotherapy, targeted therapy, and immunotherapy have significantly enhanced patient outcomes and quality of life, particularly in advanced non‐small cell lung cancer.^136^, ^137^, ^138^ Furthermore, NAT has demonstrated its role in improving resectability, delaying recurrence and progression, and prolonging survival in select lung cancer patients.^139^ However, the efficacy of these therapies varies across different populations. Molecular characteristics offer valuable insights into prognosis and therapeutic benefits,^140^ yet the clinical application of these biomarkers obtained through tissue biopsy is limited. Given the widespread use of chest CT and PET/CT in the preoperative assessment of lung cancer, radiogenomics holds promise in addressing the limitations of molecular markers, enabling better patient stratification, and facilitating treatment decision‐making.

Epidermal growth factor receptor (EGFR) gene mutations are the most prevalent targeted driver mutations in lung cancer.^141^ Constant updates are being made to EGFR‐tyrosine kinase inhibitor (TKI) targeted therapy regimens in order to combat drug resistance.^142^ The choice of therapeutic agent has always relied on the accurate identification and subtyping of EGFR mutations.^143^ Radiomic models have shown good to excellent performance in predicting EGFR mutations in lung cancer.^68^, ^144^, ^145^ DL models appear to outperform classical radiomics,^146^, ^147^ and their combination may yield even better results.^62^ Notably, an international multicenter study with a large cohort of cases developed a DL model based on the entire lung, which achieved an AUC of 0.812 in predicting EGFR status in lung cancer and successfully stratified progression‐free survival in patients treated with EGFR‐TKI. Furthermore, correlations were found between radiomic phenotypes and multiple genotypes, as well as gene pathways associated with drug resistance and cancer progression mechanisms, providing compelling evidence for the use of radiomics in predicting EGFR mutations.^148^ Taking it a step further, Wang et al.^62^ developed a radiomics‐DL joint model to determine EGFR mutation subtypes, including 19Del, L858R, and other mutations. Additionally, Yang et al.^63^ constructed a radiomic model may aid in predicting the acquired drug‐resistant mutation T790M following targeted therapy for lung cancer, suggesting the potential application of radiogenomic models in optimizing EGFR‐targeted therapy decisions.

ALK fusion is another key therapeutic target in lung cancer,^149^ and Song et al.’s CT‐based DL model yielded an AUC of 0.85 in external validation. The model also showed promising performance in predicting response to ALK‐TKI therapy, which was further validated.^66^ Regarding Kirsten rat sarcoma viral oncogene homologue (KRAS) mutation, radiomic models based on low‐dose CT scan and PET‐CT have shown good predictive performance. However, additional validation using external data is necessary.^60^, ^150^

Over the past decade, immunotherapy has emerged as a pivotal breakthrough in the treatment of lung cancer, revolutionizing the therapeutic landscape.^151^ Despite the significant advancements made in targeting immune checkpoints, particularly the programmed death receptor 1/programmed death ligand 1 (PD‐1/PD‐L1) axis, a substantial proportion of patients fail to derive benefits from PD‐1/PD‐L1 inhibitors.^151^ Although CT‐based radiomic models have been developed to predict PD‐L1 expression in non‐small cell lung cancer, with AUCs ranging from 0.66 to 0.95, their performance in external validation has been suboptimal. Nonetheless, these models have demonstrated correlations with prognosis and immunotherapy response.^57^, ^61^, ^62^, ^67^ Mu et al.^68^ constructed PET/CT‐based radiomic prediction models for PD‐L1 expression and EGFR mutation, and subsequently established treatment decision guidelines based on these models, along with Eastern Cooperative Oncology Group performance status scores. The clinical utility of these radiogenomic models was validated in external data, showcasing their effectiveness in guiding the selection of patients for TKIs and immune checkpoint inhibitors (ICIs) therapy.^68^, ^152^ In another study, a DL model was developed to predict tumor mutational burden (TMB), achieving an AUC of 0.81^69^ and was further validated in stratifying survival outcomes following immunotherapy. Additionally, several studies have demonstrated associations between radiomic subtypes and immunophenotypes, such as CD8 expression and cytolytic activity score, as well as response to immunotherapy^70^, ^153^

For some rare molecular alterations of lung cancer, radiogenomic studies are restricted by data size. A few studies tried to simultaneously predict multiple molecular subtypes through radiomics based on CT or PET/CT to reflect more realistic clinical scenarios. However, external data are still required for validation.^59^, ^154^ Notably, for the first time, one of these studies discovered that the utilization of transformer algorithms, commonly used in large language models (LLMs), for constructing radiogenomic models outperformed those based on neural networks. Furthermore, robust correlations have been observed between radiomic features, genomic features, and tumor recurrence as well as response to neoadjuvant immunotherapy in lung cancer.^58^, ^64^, ^65^ A comprehensive profiling of these radiogenomic associations contributes to improved decision‐making and the identification of novel therapeutic targets.

Recently, some studies have attempted to establish radiogenomics models to achieve better prognostic stratification of lung cancer, by combining radiomics with transcriptome, or CDK4 and TMB status.^155^, ^156^ Besides, Ju et al.^71^ investigated the interaction between genomic and radiomic features and successfully achieved noninvasive prediction of lymph node metastasis in NSCLC. These studies show the clear potential of the integration of complementary multiscale information from imaging and genes in the stratification of prognosis and treatment response in lung cancer. Notably, by liquid biopsy, de Miguel‐Perez et al.^157^ verified that dynamic expression of plasma extracellular vesicle PD‐L1 in the early stage of treatment correlated with sustained response to ICIs, and that radiogenomics modeling in conjunction with CT radiomics could further enhance the specificity, sensitivity, and accuracy of the model.

### 5.3 Breast cancer

Breast cancer is the most prevalent malignancy among women worldwide, and the characterization of its molecular markers has significantly contributed to the development of increasingly sophisticated diagnostic and treatment approaches.^158^ In particular, the utilization of NAT, immunotherapies, and novel targeted therapeutic options has underscored the importance of accessing tumor biomarkers in a noninvasive manner.^159^ Radiogenomics, which combines genetic and radiomic data, enhances genomics by providing voxel‐by‐voxel biological information for a heterogeneous tumor, enabling tailored therapy. Specifically, multiple imaging modalities, including mammography, ultrasound, MRI, CT, and PET/CT, are employed for diagnosis and treatment, thereby offering multidimensional data for accurate assessment of breast cancer.^160^

Breast cancer is commonly classified into four subtypes based on the expression of estrogen receptor (ER), progesterone receptor (PR), human epidermal growth factor receptor 2 (HER2), and Ki‐67, namely Luminal A, Luminal B, HER2‐enriched, and triple‐negative breast cancers (TNBC).^159^ Ultrasound‐based DL models have shown good performance in identifying these subtypes and distinguishing between luminal and nonluminal diseases.^81^, ^86^ However, classical radiomic features have shown limited predictive efficacy for HER2.^161^ On the other hand, mammography‐based radiomic models have shown promising results in predicting the hormone receptor and HER2 status of breast cancer.^77^, ^88^, ^162^ Zhang et al.^87^ developed a multimodal DL model using a large cohort that combined ultrasound and mammogram data. This model achieved an accuracy of 0.84 in the internal test set and an AUC of 0.92 for predicting luminal disease from nonluminal disease, significantly outperforming clinicians.

MRI is also used in radiogenomics to differentiate molecular subtypes; yet, there are few studies with large cohorts and external validation. MRI‐based radiomic models have been shown to successfully predict HER2 expression and pathologic complete response (PCR) of neoadjuvant chemotherapy (NAC) and disease‐free survival.^72^, ^163^ Some other studies have reported that radiomic models can identify ER, PR, Ki‐67 expression, or differentiate luminal A from other immunohistochemical subtypes, but all of them have only been internally or cross‐validated.^75^, ^76^, ^79^, ^164^ However, it has been observed that transfer learning can partially compensate for the lack of training data in DL, and ML algorithms may outperform DL algorithms when data are limited. Additionally, incorporating features from the peritumor region and perfusion images may improve the predictive ability of the model. Jiang et al.^74^ validated in an external dataset that MRI‐based radiomic models could identify TNBC and distinguish internal subtypes of TNBC with favorable performance. Furthermore, an association was found between peritumoral radiomic features and immune suppression and upregulated fatty acid synthesis. Fan et al.^85^ developed a radiomic model based on multiple hemodynamic subregions by referencing the unsupervised segmentation of intra‐ and extratumor regions on dynamic contrast‐enhanced images, which outperformed the simple whole‐tumor radiomic model in prediction of immunohistochemical subtypes, suggesting additional benefits obtained by delving deeper into intratumor heterogeneity from images. Interestingly, the new DL algorithm generates adversarial networks can synthesize realistic breast MRI images for training of radiomics models to predict breast cancer genotypes and mutational states.^165^Hormone receptor and/or HER2 status discordance after neoadjuvant treatment is a relatively common phenomenon and may require adjustments in post‐NAT strategies.^32^ Liu et al.^73^ constructed a radiomic model to predict post‐NAT discordance based on multimodality MRI. Although the sample size was small and external validation data were lacking, the results suggest that radiogenomics may provide guidance for retesting molecules and posttreatment alterations in the biology of cancer.

PAM50 subtyping of breast cancer, as opposed to immunohistochemical subtypes, offers superior stratification for disease progression, prognosis, and therapeutic resistance.^166^ However, the clinical application of genomic assays is limited due to their high cost. To address this issue, a cost‐effective solution was proposed in the form of a deep transfer learning model that utilizes dynamic contrast‐enhanced images to predict PAM50 subtypes.^76^ Additionally, Liang et al.^167^ attempted to establish complex many‐to‐many associations between ultrasound radiomics and genomic features to screen for key radiomic and genomic features, providing clues for biological interpretation of radiomics and targeted therapeutic decisions. Gallivanone et al.^168^ conducted a study correlating the MR radiomic phenotype of breast cancer with microRNAs, mRNAs, and regulatory networks to develop a radiomirnomic map. They found that the radiogenomic model provided better discrimination of breast cancer subtypes compared with miRNA or radiomics alone.

TIME is a crucial element in the progression and metastasis of breast cancer.^169^ PD‐L1 and tumor‐infiltrating lymphocytes are strongly associated with immune evasion by tumors and serve as vital biomarkers for the effectiveness of ICIs.^170^ MRI‐based radiomic models have been shown to predict PD‐L1 expression, tumor microenvironment phenotypes based on immune cell infiltration and omics.^78^, ^80^, ^82^, ^83^, ^171^ Lv et al.^84^ screened for genome‐related imaging features to construct interpretable imaging phenotypes that could predict different molecular features, including hormone receptor, epithelial growth factor receptor, and immune checkpoint protein expression. While still in the early stages, radiogenomics shows potential in enhancing noninvasive preoperative evaluation of the TIME and supporting the clinical implementation of immunotherapy.

Prediction of NAC response in breast cancer is a hot spot in radiogenomics. The addition of baseline MRI radiomics to molecular markers did not significantly improve the prediction of PCR after NAC.^172^ However, radiomic models based on longitudinal MRI have shown improved predictive performance in comparison with molecular subtyping.^173^, ^174^ Similarly, a radiogenomic model combining five variant allele frequency features of nonsynonymous mutation sites and baseline MRI was able to predict PCR to NAC in TNBC patients, and a potential relationship was found between two high‐frequency mutations and epidoxorubicin resistance.^91^ Huang et al.^175^ developed a radiogenomic model incorporating MRI features, ER expression, and Ki‐67, which achieved an AUC of 0.94 in predicting tumor shrinkage patterns after NAC and maintained good predictive performance across different molecular subtypes. Recently, Radiogenomic models that united radiomics and transcriptomics were demonstrated to predict axillary lymph node metastasis as well as and response to drug therapy, while gene pathway enrichment analyses showed significant differences in signaling pathway activation across risk groups.^93^ DCE‐based radiomics, reflecting intratumor and peritumor hemodynamic heterogeneity, in conjunction with genomics to constitutes a radiogenomics model also showed significant potential in predicting PCR and poor prognosis in TNBC patients.^92^These studies demonstrated the significant potential of radiogenomic models in predicting treatment response. Furthermore, radiogenomic models that incorporate CT, molecular subtyping, and clinical features from multicenter cohorts have shown promise in predicting immunotherapy response in breast cancer patients.^90^

### 5.4 Other cancers

In recent years, significant advancements have been achieved in the field of radiogenomics for various types of cancers. For example, CT‐based radiomic models have been developed to predict specific gene mutations in clear cell renal cell carcinoma (ccRCC), such as VHL, Polybromo‐1 mutation, and Loss 9p21.3.^102^, ^176^ Radiomic models have also been used to differentiate omics‐based lipid metabolism or TIME subtypes and correlate them with patient survival.^100^, ^101^Unsupervised clustering of radiomic and transcriptomic features of ccRCC allows for the identification of intrinsic subtypes which exhibit unique clinicopathological, prognostic, immunological, and molecular features, and this is expected to facilitate personalized diagnostic and treatment decisions.^177^ Besides, It has been found that a radiogenomic model provided more accurate predictions of overall survival in kidney cancer compared with using radiomics alone.^102^ Additionally, CT‐based DL models have been leveraged to predict the mutation status of the RAS gene and DNA mismatch repair in colorectal cancer.^94^, ^95^ Furthermore, a radiomic model based on PET images has shown promise in predicting tumor mutation burden and its correlation with prognosis.^96^ Zhong et al.^99^ showed that radiomic features were associated with tumor genome subcloning, and radiogenomic signatures could serve as independent predictors of prognosis in patients with colorectal cancer. In the case of liver metastases commonly found in colorectal cancer, radiomic scores have been used to determine RAS and BRAF mutation status, as well as CD73 expression.^97^, ^98^ For thyroid cancer, CT radiomic models could predict the status of cytokeratin 19, galectin 3, thyroperoxidase, and high‐molecular‐weight cytokeratin.^178^ Recent studies showed radiomics features of prostate cancer and its bone metastases correlate with liquid biopsy monitoring of CTCs, free DNA, and genes related to apoptosis, hypoxia, and androgen receptor expression.^103^, ^104^ The potential of radiogenomics in cancer diagnosis and prognosis is immense; however, further works are warranted to explore its full scope, applicability, and clinical application.

## 6 CURRENT CHALLENGES AND FUTURE DIRECTIONS

Despite the reported successes of radiogenomics, several limitations and hurdles need to be addressed before widespread clinical adoption.

### 6.1 Stability and repeatability

The stability and repeatability of radiogenomic models is a key factor in their clinical translation. However, radiomic features are prone to instability caused by various factors, even though they sensitively characterize tumor heterogeneity.^179^ Moreover, most of the results are based on retrospective studies with small sample sizes, which introduces bias in the data inclusion and analysis process. This makes it difficult to apply the findings to other centers and actual clinical scenarios. To mitigate these issues, standardization algorithms can be applied to reduce variations in medical images between machines and centers.^180^ Besides, selecting stable features for constructing radiogenomic models is crucial.^181^ In addition, improving the transparency and explainability of radiogenomics models helps to detect model hallucinations and biases and make adjustments before they are put into complex clinical scenarios.^182^

More importantly, training the model using representative heterogeneous data with rigorous external validation rigorous external validation, especially in multicenter real‐world settings. We also need to assessing the general applicability of the model across different populations, because potential variation may exist in the efficacy of radiogenomic models due to differences in training data or biological factors between populations, such as race and gender.^183^ This variation can potentially lead to social injustices. Therefore, it is important to effectively control bias in the training data and adequately assess and specify the scope of model applicability.^184^ Finally, the open source of radiogenomics modeling code and the sharing of resources may facilitate the reproduction and further validation of modeling results by other researchers.

### 6.2 Explainability and interpretability

Although there are arguments that explainability of artificial intelligence (AI) is impractical, while rigorous validation of model efficacy and robustness is even more important.^185^ However, we still believe that explainability of medical AI is critical and worth working towards. As radiogenomics models have become progressively complex to achieve greater predictive power, explainability decreased. DL is considered a “black box,” causing concern that it may make mistakes in complex clinical scenarios that exceed expectations, which can have a significant impact on medical decision‐making. As a tool, it is important for medical professionals to understand the scope of its use, the mistakes it can make, and the corresponding solutions.^186^ In radiogenomics, there are different ways to achieve explainability and interpretability of models. One is in the feature extraction stage, where algorithms are used to extract features that reflect specific biological information about the cancer, and the association of this information with the prediction task is comprehensible, making the models constructed from features carrying biological information highly interpretable. For example, traditional radiomic features are used to quantify tumor morphology, signal intensity, and heterogeneity information,^187^ and brain network features reflect damage to white matter fiber tracts.^16^ DL model construction guided by biological information also has higher interpretability than direct models.^188^ Second, some researchers have evaluated the model results by feature attribution (e.g., Shapley Additive Explanation), attention‐based (e.g., Class Activation Map), and example‐based methods to verify whether the model has an understandable inference mechanism.^120^, ^189^ These initiatives allow researchers to detect and control how radiogenomics models are performed, ensuring that they are always aligned with our clinical goals.

### 6.3 Data hunger

Radiogenomics increasingly craves for adequate matched images and multiomics data support. Molecular data on cancer are inherently limited due to its clinical importance, acquisition costs, and patient affordability. Fortunately, the availability of large‐volume omics and image data through increasing open datasets in recent years has facilitated the development of radiogenomics.^190^ The Cancer Imaging Archive (TCIA) and The Cancer Genome Atlas (TCGA) are leading the way. TCIA is an extensive repository of medical images, including CT, MRI, and digital histopathology images, specifically curated for cancer research, while TCGA has cataloged genomic, epigenomic, transcriptomic, and proteomic data from thousands of cancer patients across more than 30 different cancer types. Importantly, their data are correlated. For many of the patients included in TCGA, corresponding imaging data are available in TCIA, allowing researchers to correlate omics and other molecular profiles with radiomics. TCIA and TCGA have supported numerous high‐impact radiogenomic studies that have led to new insights into cancer biology, improved diagnostic techniques, and the development of targeted therapies. Additionally, the privacy and data protection efforts of both TCIA and TCGA are exemplary for other data‐sharing projects.^191^, ^192^, ^193^

Besides, cross‐institutional and even international collaborations are also significant in providing sufficiently heterogeneous data. Yet the distribution of costs and benefits of research and concerns about privacy are major obstacles. The establishment of equitable and mutually beneficial cooperative agreements to share research data, equipment and scientific findings facilitates reliable and stable partnerships. By adopting data anonymization techniques, such as differential privacy and federated learning, potential risks related to patient privacy during data sharing can be minimized.^194^, ^195^, ^196^

Additionally, even with widely used imaging modalities, imaging data can be incomplete due to inconsistent imaging protocols or poor data management. Furthermore, the imbalance in data exacerbates the impact of overall insufficient data, making it challenging to train effective predictive models. Data augmentation algorithms and generative AI can be used to generate high‐quality synthetic data to compensate for unbalanced or incomplete data, enabling more effective model training.^197^, ^198^ Moreover, the utilization of DL algorithms such as transfer learning and self‐supervised learning holds the potential to fully leverage pretrained base models or large amounts of unsupervised data, resulting in high‐quality predictive models despite limited target samples.^15^, ^199^, ^200^

### 6.4 Spatiotemporal registration in radiogenomics

The spatial and temporal genetic heterogeneity of cancers has been extensively studied and well documented. While imaging has shown potential in identifying these heterogeneities in some preliminary studies, there are still significant challenges in the field of radiogenomics, particularly in obtaining sufficient spatially and temporally based molecular data and aligning them with images. The ability to perform virtual biopsies of multiple regions or lesions and continuously monitor molecular changes within tumors is still a major challenge in terms of experimental design and execution. Fortunately, advances in spatiotemporal omics and liquid biopsy technology hold promise for the acquisition of spatiotemporal molecular data of cancers, and may present significant opportunities for decoding of spatiotemporal heterogeneity of cancers using radiogenomics.

### 6.5 Diversity of radiology

The key to radiogenomics lies in identifying valuable features that can accurately predict clinical outcomes. Although DL models automatically learn representations of image features and show superior results to classical radiomics in various tasks, hand‐crafted radiomics carry advantages including less data dependency, the ability to incorporate clinical prior knowledge, and higher interpretability. By integrating DL with radiomics, we can fully leverage the potential of imaging data. Furthermore, data obtained from multiple anatomical scales and multimodalities can provide multidimensional features that enable the characterization of cancer molecules and prognosis, with the potential to amplify the signals of specific molecules and microenvironment components.

Medical images commonly used are typically designed and generated for human visual interpretation. However, when it comes to radiomics, it is important to consider the differences between computer vision and human vision. These traditional forms of input may not always be optimal for achieving accurate and consistent outputs. A notable example is the need for quantization processing on medical images in classical radiomics, which is often performed prior to feature extraction. In recent years, the emergence of MR fingerprint technology has enabled the translation of current visual and qualitative MRI diagnostic criteria into a quantitative acquisition and analysis framework.^201^ Additionally, raw data obtained from imaging equipment may contain biological information that can be utilized for computer processing, as opposed to medical images that have undergone graphical manipulations to enhance readability.^50^ By altering the approach to image collection and processing, making them more suitable for ML, we can further advance the development of radiogenomics.

### 6.6 Multimodal AI

Finally, the integration and analysis of multiple types of medical data are considered crucial for advancing precision oncology. It is necessary to introduce multimodal ML algorithms capable of handling various data types to replace the linear regression methods commonly used for combining radiomic and molecular data. Additionally, the medical community has been inspired by LLMs and derived multimodal AI agents, which have gained significant attention.^202^, ^203^ In recent studies, multimodal LLMs can simultaneously interpret text and images to generate reports, closely mimicking current diagnostic pathways in radiology.^204^It is foreseeable that multimodal LLMs can be integrated in a variety of real‐world medical support scenarios including preoperative biomarker profiling of tumors with images, and postoperative clinical decision making based on radiogenomics, in a natural language‐based interaction. This process can be based on specific predictive capabilities obtained after targeted training, or the ability to call appropriate validated radiogenomics models autonomously, as possessed by the AI agent. In the future, AI agents based on medical foundation models may represent an ideal form of efficient automated precision medicine. These agents can assist in integrating patients' medical data and invoking appropriate validated specialized models for tasks such as patient counseling, disease classification, prognosis, and decision‐making.^205^

## 7 CONCLUSIONS AND PROSPECTS

Recent high‐quality studies provide compelling evidence of its clinical utility by bridging fundamental research findings with precision medicine applications. Although there are still non‐negligible problems to overcome in radiogenomics. For example, the massive amounts of high‐quality imaging and biomolecular data for constructing strong enough radiogenomics models are still insufficient. Accurate matching of imaging genomics and multiomics data in time and space still faces great challenges at the technical level.

Nationally driven large‐scale public databases and collaborative projects, as well as the development of generative AI for virtual data synthesis, are expected to greatly facilitated development by consistently providing access to heterogeneous imaging and genomic data at scale. Incorporating structural, functional, and molecular radiomic at various anatomical scale levels and multiomics information from tissue and liquid samples enables more comprehensive and time‐critical tumor characterization, understanding, and prediction, providing determining basis for precision oncology. Advances in AI algorithms, such as attention mechanisms, transfer learning, and self‐supervised learning, are striving to utilize variable‐quality but highly accessible data to break through the limitation of insufficient high‐quality data. What is more, with the help of multimodal LLMs, it is expected to facilitate the efficient implementation of the latest results of radiogenomics into the existing clinical workflow.

Above all, standards for rigorous model evaluation are essential to assess validity, generalizability and clinical applicability. The promise of radiogenomics still warrants more real‐world validation and evaluation of its efficacy in diverse populations to ensure that these technologies are equitably delivering benefits to patients. Only through such evaluation can radiogenomic models be reliably applied to improve patient outcomes. Continued research efforts are moving the field closer to realizing radiogenomics' full potential for precision oncology.

## AUTHOR CONTRIBUTIONS

We confirm contribution to the paper as follows. *Study conception*: Bin Zhang and Shuixing Zhang. *Literature review*: Wenle He, Bin Zhang, Shuixing Zhang, Xuewei Wu, Wenhui Huang, and Lu Zhang. *Draft manuscript preparation*: Wenhui Huang, Bin Zhang, and Shuixing Zhang. All authors reviewed the results and approved the final version of the manuscript.

## CONFLICT OF INTEREST STATEMENT

The authors declare that they have no conflict of interest.

## ETHICS STATEMENT

Not applicable.

## Data Availability

Not applicable.

## References

[1] Sung H , Ferlay J , Siegel RL , et al. Global cancer statistics 2020: GLOBOCAN estimates of incidence and mortality worldwide for 36 cancers in 185 countries. CA Cancer J Clin. 2021;71(3):209‐249. 33538338 10.3322/caac.21660

[2] Stratton MR , Campbell PJ , Futreal PA . The cancer genome. Nature. 2009;458(7239):719‐724. 19360079 10.1038/nature07943 PMC2821689

[3] De Visser KE , Joyce JA . The evolving tumor microenvironment: from cancer initiation to metastatic outgrowth. Cancer Cell. 2023;41(3):374‐403. 36917948 10.1016/j.ccell.2023.02.016

[4] Akhoundova D , Rubin MA . Clinical application of advanced multi‐omics tumor profiling: shaping precision oncology of the future. Cancer Cell. 2022;40(9):920‐938. 36055231 10.1016/j.ccell.2022.08.011

[5] Gillies RJ , Kinahan PE , Hricak H . Radiomics: images are more than pictures, they are data. Radiology. 2016;278(2):563‐577. 26579733 10.1148/radiol.2015151169 PMC4734157

[6] Lambin P , Leijenaar RTH , Deist TM , et al. Radiomics: the bridge between medical imaging and personalized medicine. Nat Rev Clin Oncol. 2017;14(12):749‐762. 28975929 10.1038/nrclinonc.2017.141

[7] Pinker K , Chin J , Melsaether AN , Morris EA , Moy L . Precision medicine and radiogenomics in breast cancer: new approaches toward diagnosis and treatment. Radiology. 2018;287(3):732‐747. 29782246 10.1148/radiol.2018172171

[8] Hartmann K , Sadée CY , Satwah I , Carrillo‐Perez F , Gevaert O . Imaging genomics: data fusion in uncovering disease heritability. Trends Mol Med. 2023;29(2):141‐151. 36470817 10.1016/j.molmed.2022.11.002 PMC10507799

[9] Li ZC , Yan J , Zhang S , et al. Glioma survival prediction from whole‐brain MRI without tumor segmentation using deep attention network: a multicenter study. Eur Radiol. 2022;32(8):5719‐5729. 35278123 10.1007/s00330-022-08640-7

[10] Wang S , Yu H , Gan Y , et al. Mining whole‐lung information by artificial intelligence for predicting EGFR genotype and targeted therapy response in lung cancer: a multicohort study. Lancet Digit Health. 2022;4(5):e309‐e319. 35341713 10.1016/S2589-7500(22)00024-3

[11] Van Griethuysen JJM , Fedorov A , Parmar C , et al. Computational radiomics system to decode the radiographic phenotype. Cancer Res. 2017;77(21):e104‐e107. 29092951 10.1158/0008-5472.CAN-17-0339 PMC5672828

[12] Van der Voort SR , Incekara F , Wijnenga MMJ , et al. Combined molecular subtyping, grading, and segmentation of glioma using multi‐task deep learning. Neuro‐Oncol. 2023;25(2):279‐289. 35788352 10.1093/neuonc/noac166 PMC9925710

[13] Decuyper M , Bonte S , Deblaere K , Van Holen R . Automated MRI based pipeline for segmentation and prediction of grade, IDH mutation and 1p19q co‐deletion in glioma. Comput Med Imaging Graph. 2021;88:101831. October 2020. 33482430 10.1016/j.compmedimag.2020.101831

[14] Zhang B , Wu X , Zhang S , et al. Biologically interpretable multi‐task deep learning pipeline predicts molecular alterations, grade, and prognosis in glioma patients. Published online February 20, 2024. 10.1038/s41698-024-00670-2 PMC11329669 39152182

[15] Wei Y , Chen X , Zhu L , et al. Multi‐modal learning for predicting the genotype of glioma. IEEE Trans Med Imaging. 2023. PP. 10.1109/TMI.2023.3244038 37022918

[16] Yang N , Xiao X , Gu G , et al. Diffusion MRI‐based connectomics features improve the noninvasive prediction of H3K27M mutation in brainstem gliomas. Radiother Oncol. 2023;186:109789. 37414255 10.1016/j.radonc.2023.109789

[17] Ismail M , Prasanna P , Bera K , et al. Radiomic deformation and textural heterogeneity (R‐depth) descriptor to characterize tumor field effect: application to survival prediction in glioblastoma. IEEE Trans Med Imaging. 2022;41(7):1764‐1777. 35108202 10.1109/TMI.2022.3148780 PMC9575333

[18] Liu X , Zhang Q , Li J , et al. Coordinatized lesion location analysis empowering ROI‐based radiomics diagnosis on brain gliomas. Eur Radiol. 2023;33(12):8776‐8787. 10.1007/s00330-023-09871-y 37382614

[19] Baysoy A , Bai Z , Satija R , Fan R . The technological landscape and applications of single‐cell multi‐omics. Nat Rev Mol Cell Biol. 2023;24(10):695‐713. 37280296 10.1038/s41580-023-00615-w PMC10242609

[20] Nam AS , Chaligne R , Landau DA . Integrating genetic and non‐genetic determinants of cancer evolution by single‐cell multi‐omics. Nat Rev Genet. 2021;22(1):3‐18. 32807900 10.1038/s41576-020-0265-5 PMC8450921

[21] Siravegna G , Marsoni S , Siena S , Bardelli A . Integrating liquid biopsies into the management of cancer. Nat Rev Clin Oncol. 2017;14(9):531‐548. 28252003 10.1038/nrclinonc.2017.14

[22] Passaro A , Al Bakir M , Hamilton EG , et al. Cancer biomarkers: emerging trends and clinical implications for personalized treatment. Cell. 2024;187(7):1617‐1635. 38552610 10.1016/j.cell.2024.02.041 PMC7616034

[23] Li MM , Cottrell CE , Pullambhatla M , et al. Assessments of somatic variant classification using the Association for Molecular Pathology/American Society of Clinical Oncology/College of American Pathologists Guidelines: a Report from the Association for Molecular Pathology. J Mol Diagn. 2023;25(2):69‐86. 36503149 10.1016/j.jmoldx.2022.11.002

[24] Jose A , Kulkarni P , Thilakan J , et al. Integration of pan‐omics technologies and three‐dimensional in vitro tumor models: an approach toward drug discovery and precision medicine. Mol Cancer. 2024;23(1):50. 38461268 10.1186/s12943-023-01916-6 PMC10924370

[25] Bressan D , Battistoni G , Hannon GJ . The dawn of spatial omics. Science. 2023;381(6657):eabq4964. 37535749 10.1126/science.abq4964 PMC7614974

[26] Nikanjam M , Kato S , Kurzrock R . Liquid biopsy: current technology and clinical applications. J Hematol Oncol. 2022;15(1):131. 36096847 10.1186/s13045-022-01351-y PMC9465933

[27] Wang H , Zhang Y , Zhang H , et al. Liquid biopsy for human cancer: cancer screening, monitoring, and treatment. Medcomm. 2024;5(6):e564. 38807975 10.1002/mco2.564 PMC11130638

[28] Holder AM , Dedeilia A , Sierra‐Davidson K , et al. Defining clinically useful biomarkers of immune checkpoint inhibitors in solid tumours. Nat Rev Cancer. 2024;24(7):498‐512. 38867074 10.1038/s41568-024-00705-7

[29] Kagawa Y , Smith JJ , Fokas E , et al. Future direction of total neoadjuvant therapy for locally advanced rectal cancer. Nat Rev Gastroenterol Hepatol. 2024;21(6):444‐455. 38485756 10.1038/s41575-024-00900-9 PMC11588332

[30] Dagogo‐Jack I , Shaw AT . Tumour heterogeneity and resistance to cancer therapies. Nat Rev Clin Oncol. 2018;15(2):81‐94. 29115304 10.1038/nrclinonc.2017.166

[31] Liang Y , Zhang H , Song X , Yang Q . Metastatic heterogeneity of breast cancer: molecular mechanism and potential therapeutic targets. Semin Cancer Biol. 2020;60:14‐27. 31421262 10.1016/j.semcancer.2019.08.012

[32] Zhu S , Lu Y , Fei X , Shen K , Chen X . Pathological complete response, category change, and prognostic significance of HER2‐low breast cancer receiving neoadjuvant treatment: a multicenter analysis of 2489 cases. Br J Cancer. 2023;129(8):1274‐1283. 37604930 10.1038/s41416-023-02403-x PMC10575949

[33] Zhang L , Wang R , Gao J , et al. A novel MRI‐based deep learning networks combined with attention mechanism for predicting CDKN2A/B homozygous deletion status in IDH‐mutant astrocytoma. Eur Radiol. 2024;34(1):391‐399. 10.1007/s00330-023-09944-y 37553486

[34] Ma C , Wang L , Song D , et al. Multimodal‐based machine learning strategy for accurate and non‐invasive prediction of intramedullary glioma grade and mutation status of molecular markers: a retrospective study. BMC Med. 2023;21(1):198. 37248527 10.1186/s12916-023-02898-4 PMC10228074

[35] Duan J , Zhang Z , Chen Y , et al. Imaging phenotypes from MRI for the prediction of glioma immune subtypes from RNA sequencing: a multicenter study. Mol Oncol. 2023;17(4):629‐646. 36688633 10.1002/1878-0261.13380 PMC10061289

[36] Zhang L , Pan H , Liu Z , et al. Multicenter clinical radiomics‐integrated model based on [(18)F]FDG PET and multi‐modal MRI predict ATRX mutation status in IDH‐mutant lower‐grade gliomas. Eur Radiol. 2023;33(2):872‐883. 35984514 10.1007/s00330-022-09043-4

[37] Sha Y , Yan Q , Tan Y , Wang X , Zhang H , Yang G . Prediction of the molecular subtype of IDH mutation combined with MGMT promoter methylation in gliomas via radiomics based on preoperative MRI. Cancers. 2023;15(5). 10.3390/cancers15051440 PMC10001198 36900232

[38] Kim BH , Lee H , Choi KS , et al. Validation of MRI‐based models to predict MGMT promoter methylation in gliomas: brats 2021 radiogenomics challenge. Cancers. 2022;14(19). 10.3390/cancers14194827 PMC9562637 36230750

[39] Cluceru J , Interian Y , Phillips JJ , et al. Improving the noninvasive classification of glioma genetic subtype with deep learning and diffusion‐weighted imaging. Neuro‐Oncol. 2022;24(4):639‐652. 34653254 10.1093/neuonc/noab238 PMC8972294

[40] Wu C , Zheng H , Li J , et al. MRI‐based radiomics signature and clinical factor for predicting H3K27M mutation in pediatric high‐grade gliomas located in the midline of the brain. Eur Radiol. 2022;32(3):1813‐1822. 34655310 10.1007/s00330-021-08234-9

[41] Soldatelli MD , Namdar K , Tabori U , et al. Identification of multiclass pediatric low‐grade neuroepithelial tumor molecular subtype with ADC MR imaging and machine learning. AJNR Am J Neuroradiol. 2024;45(6):753‐760. 38604736 10.3174/ajnr.A8199 PMC11288584

[42] Li Y , Wei D , Liu X , et al. Molecular subtyping of diffuse gliomas using magnetic resonance imaging: comparison and correlation between radiomics and deep learning. Eur Radiol. 2022;32(2):747‐758. 34417848 10.1007/s00330-021-08237-6

[43] Zhuo Z , Qu L , Zhang P , et al. Prediction of H3K27M‐mutant brainstem glioma by amide proton transfer‐weighted imaging and its derived radiomics. Eur J Nucl Med Mol Imaging. 2021;48(13):4426‐4436. 34131804 10.1007/s00259-021-05455-4

[44] Li Z , Kaiser L , Holzgreve A , et al. Prediction of tertp‐mutation status in IDH‐wildtype high‐grade gliomas using pre‐treatment dynamic [18F]FET PET radiomics. Eur J Nucl Med Mol Imaging. 2021;48(13):4415‐4425. 34490493 10.1007/s00259-021-05526-6 PMC8566644

[45] Beig N , Singh S , Bera K , et al. Sexually dimorphic radiogenomic models identify distinct imaging and biological pathways that are prognostic of overall survival in glioblastoma. Neuro‐Oncol. 2021;23(2):251‐263. 33068415 10.1093/neuonc/noaa231 PMC7906064

[46] Yan J , Zhang B , Zhang S , et al. Quantitative MRI‐based radiomics for noninvasively predicting molecular subtypes and survival in glioma patients. NPJ Precis Oncol. 2021;5(1):72. 34312469 10.1038/s41698-021-00205-z PMC8313682

[47] Qian J , Herman MG , Brinkmann DH , et al. Prediction of MGMT status for glioblastoma patients using radiomics feature extraction from 18F‐DOPA‐PET imaging. Int J Radiat Oncol Biol Phys. 2020;108(5):1339‐1346. 32634544 10.1016/j.ijrobp.2020.06.073 PMC7680434

[48] Park JE , Kim HS , Park SY , et al. Prediction of core signaling pathway by using diffusionand perfusion‐based mri radiomics and next‐generation sequencing in isocitrate dehydrogenase wild‐type glioblastoma. Radiology. 2020;294(2):388‐397. 31845844 10.1148/radiol.2019190913

[49] Buda M , Albadawy EA , Saha A , Mazurowski MA . Deep radiogenomics of lower‐grade gliomas: convolutional neural networks predict tumor genomic subtypes using MR images. Radiol Artif Intell. 2020;2(1):e180050. 33937809 10.1148/ryai.2019180050 PMC8017403

[50] Choi KS , Choi SH , Jeong B . Prediction of IDH genotype in gliomas with dynamic susceptibility contrast perfusion MR imaging using an explainable recurrent neural network. Neuro‐Oncol. 2019;21(9):1197‐1209. 31127834 10.1093/neuonc/noz095 PMC7594560

[51] Zinn PO , Singh SK , Kotrotsou A , et al. A coclinical radiogenomic validation study: conserved magnetic resonance radiomic appearance of periostin‐expressing glioblastoma in patients and xenograft models. Clin Cancer Res. 2018;24(24):6288‐6299. 30054278 10.1158/1078-0432.CCR-17-3420 PMC6538261

[52] Yan J , Sun Q , Tan X , et al. Image‐based deep learning identifies glioblastoma risk groups with genomic and transcriptomic heterogeneity: a multi‐center study. Eur Radiol. 2023;33(2):904‐914. 36001125 10.1007/s00330-022-09066-x

[53] Yan J , Zhao Y , Chen Y , et al. Deep learning features from diffusion tensor imaging improve glioma stratification and identify risk groups with distinct molecular pathway activities. Ebiomedicine. 2021;72:103583. 34563923 10.1016/j.ebiom.2021.103583 PMC8479635

[54] Wang J , Zheng X , Zhang J , et al. An MRI‐based radiomics signature as a pretreatment noninvasive predictor of overall survival and chemotherapeutic benefits in lower‐grade gliomas. Eur Radiol. 2021;31(4):1785‐1794. 33409797 10.1007/s00330-020-07581-3

[55] Guo J , Fathi Kazerooni A , Toorens E , et al. Integrating imaging and genomic data for the discovery of distinct glioblastoma subtypes: a joint learning approach. Sci Rep. 2024;14(1):4922. 38418494 10.1038/s41598-024-55072-y PMC10902376

[56] Sun Y , Zhang Y , Gan J , et al. Comprehensive quantitative radiogenomic evaluation reveals novel radiomic subtypes with distinct immune pattern in glioma. Comput Biol Med. 2024;177:108636. 38810473 10.1016/j.compbiomed.2024.108636

[57] Chen M , Lu H , Copley SJ , et al. A novel radiogenomics biomarker for predicting treatment response and pneumotoxicity from programmed cell death protein or ligand‐1 inhibition immunotherapy in NSCLC. J Thorac Oncol. 2023;18(6):718‐730. 36773776 10.1016/j.jtho.2023.01.089

[58] Ju HM , Kim BC , Lim I , Byun BH , Woo SK . Estimation of an image biomarker for distant recurrence prediction in NSCLC using proliferation‐related genes. Int J Mol Sci. 2023;24(3). 10.3390/ijms24032794 PMC9917349 36769108

[59] Shao J , Ma J , Zhang S , et al. Radiogenomic system for non‐invasive identification of multiple actionable mutations and PD‐L1 expression in non‐small cell lung cancer based on CT images. Cancers. 2022;14(19):1‐18. 10.3390/cancers14194823 PMC9563625 36230746

[60] Shiri I , Amini M , Nazari M , et al. Impact of feature harmonization on radiogenomics analysis: prediction of EGFR and KRAS mutations from non‐small cell lung cancer PET/CT images. Comput Biol Med. 2022;142:105230. 35051856 10.1016/j.compbiomed.2022.105230

[61] Wang C , Ma J , Shao J , et al. Non‐invasive measurement using deep learning algorithm based on multi‐source features fusion to predict PD‐L1 expression and survival in NSCLC. Front Immunol. 2022;13:828560. 35464416 10.3389/fimmu.2022.828560 PMC9022118

[62] Wang C , Ma J , Shao J , et al. Predicting EGFR and PD‐L1 status in NSCLC patients using multitask AI system based on CT images. Front Immunol. 2022;13:813072. 35250988 10.3389/fimmu.2022.813072 PMC8895233

[63] Yang X , Fang C , Li C , et al. Can CT radiomics detect acquired T790M mutation and predict prognosis in advanced lung adenocarcinoma with progression after first‐ or second‐generation EGFR tkis? Front Oncol. 2022;12(July):1‐10. 10.3389/fonc.2022.904983 PMC9300753 35875167

[64] She Y , He B , Wang F , et al. Deep learning for predicting major pathological response to neoadjuvant chemoimmunotherapy in non‐small cell lung cancer: a multicentre study. Ebiomedicine. 2022;86:104364. 36395737 10.1016/j.ebiom.2022.104364 PMC9672965

[65] Perez‐Johnston R , Araujo‐Filho JA , Connolly JG , et al. CT‐based radiogenomic analysis of clinical stage I lung adenocarcinoma with histopathologic features and oncologic outcomes. Radiology. 2022;303(3):664‐672. 35230187 10.1148/radiol.211582 PMC9131171

[66] Song Z , Liu T , Shi L , et al. The deep learning model combining CT image and clinicopathological information for predicting ALK fusion status and response to ALK‐TKI therapy in non‐small cell lung cancer patients. Eur J Nucl Med Mol Imaging. 2021;48(2):361‐371. 32794105 10.1007/s00259-020-04986-6

[67] Tian P , He B , Mu W , et al. Assessing PD‐L1 expression in non‐small cell lung cancer and predicting responses to immune checkpoint inhibitors using deep learning on computed tomography images. Theranostics. 2021;11(5):2098‐2107. 33500713 10.7150/thno.48027 PMC7797686

[68] Mu W , Jiang L , Zhang JY , et al. Non‐invasive decision support for NSCLC treatment using PET/CT radiomics. Nat Commun. 2020;11(1):5228. 33067442 10.1038/s41467-020-19116-x PMC7567795

[69] He B , Dong D , She Y , et al. Predicting response to immunotherapy in advanced non‐small‐cell lung cancer using tumor mutational burden radiomic biomarker. J Immunother Cancer. 2020;8(2). 10.1136/jitc-2020-000550 PMC7342823 32636239

[70] Park C , Na KJ , Choi H , et al. Tumor immune profiles noninvasively estimated by FDG PET with deep learning correlate with immunotherapy response in lung adenocarcinoma. Theranostics. 2020;10(23):10838‐10848. 32929383 10.7150/thno.50283 PMC7482798

[71] Ju H , Kim K , Kim BI , Woo SK . Graph neural network model for prediction of non‐small cell lung cancer lymph node metastasis using protein‐protein interaction network and (18)F‐FDG PET/CT radiomics. Int J Mol Sci. 2024;25(2). 10.3390/ijms25020698 PMC10815846 38255770

[72] Guo Y , Xie X , Tang W , et al. Noninvasive identification of HER2‐low‐positive status by MRI‐based deep learning radiomics predicts the disease‐free survival of patients with breast cancer. Eur Radiol. 2024;34(2):899‐913. 10.1007/s00330-023-09990-6 37597033

[73] Liu HQ , Lin SY , Song YD , et al. Machine learning on MRI radiomic features: identification of molecular subtype alteration in breast cancer after neoadjuvant therapy. Eur Radiol. 2023;33(4):2965‐2974. 36418622 10.1007/s00330-022-09264-7

[74] Jiang L , You C , Xiao Y , et al. Radiogenomic analysis reveals tumor heterogeneity of triple‐negative breast cancer. Cell Rep Med. 2022;3(7):100694. 35858585 10.1016/j.xcrm.2022.100694 PMC9381418

[75] Lee JY , Lee KS , Seo BK , et al. Radiomic machine learning for predicting prognostic biomarkers and molecular subtypes of breast cancer using tumor heterogeneity and angiogenesis properties on MRI. Eur Radiol. 2022;32(1):650‐660. 34226990 10.1007/s00330-021-08146-8

[76] Ming W , Li F , Zhu Y , et al. Predicting hormone receptors and PAM50 subtypes of breast cancer from multi‐scale lesion images of DCE‐MRI with transfer learning technique. Comput Biol Med. 2022;150:106147. 36201887 10.1016/j.compbiomed.2022.106147

[77] Petrillo A , Fusco R , Di Bernardo E , et al. Prediction of breast cancer histological outcome by radiomics and artificial intelligence analysis in contrast‐enhanced mammography. Cancers. 2022;14(9). 10.3390/cancers14092132 PMC9102628 35565261

[78] Jeon SH , Kim SW , Na K , Seo M , Sohn YM , Lim YJ . Radiomic models based on magnetic resonance imaging predict the spatial distribution of CD8+ tumor‐infiltrating lymphocytes in breast cancer. Front Immunol. 2022;13(December):1‐12. 10.3389/fimmu.2022.1080048 PMC9806253 36601118

[79] Fan M , Yuan C , Huang G , et al. A framework for deep multitask learning with multiparametric magnetic resonance imaging for the joint prediction of histological characteristics in breast cancer. IEEE J Biomed Health Inform. 2022;26(8):3884‐3895. 35635826 10.1109/JBHI.2022.3179014

[80] Su GH , Xiao Y , Jiang L , et al. Radiomics features for assessing tumor‐infiltrating lymphocytes correlate with molecular traits of triple‐negative breast cancer. J Transl Med. 2022;20(1):471. 36243806 10.1186/s12967-022-03688-x PMC9571493

[81] Jiang M , Zhang D , Tang SCC , et al. Deep learning with convolutional neural network in the assessment of breast cancer molecular subtypes based on US images: a multicenter retrospective study. Eur Radiol. 2021;31(6):3673‐3682. 33226454 10.1007/s00330-020-07544-8

[82] Han X , Cao W , Wu L , Liang C . Radiomics assessment of the tumor immune microenvironment to predict outcomes in breast cancer. Front Immunol. 2022;12:1‐9. January. 10.3389/fimmu.2021.773581 PMC8761791 35046937

[83] Lo Gullo R , Wen H , Reiner JS , et al. Assessing pd‐l1 expression status using radiomic features from contrast‐enhanced breast mri in breast cancer patients: initial results. Cancers. 2021;13(24):1‐13. 10.3390/cancers13246273 PMC8699819 34944898

[84] Lv T , Hong X , Liu Y , et al. AI‐powered interpretable imaging phenotypes noninvasively characterize tumor microenvironment associated with diverse molecular signatures and survival in breast cancer. Comput Methods Programs Biomed. 2024;243:107857. 37865058 10.1016/j.cmpb.2023.107857

[85] Fan M , Zhang P , Wang Y , et al. Radiomic analysis of imaging heterogeneity in tumours and the surrounding parenchyma based on unsupervised decomposition of DCE‐MRI for predicting molecular subtypes of breast cancer. Eur Radiol. 2019;29(8):4456‐4467. 30617495 10.1007/s00330-018-5891-3

[86] Quan MY , Huang YX , Wang CY , Zhang Q , Chang C , Zhou SC . Deep learning radiomics model based on breast ultrasound video to predict HER2 expression status. Front Endocrinol. 2023;14:1144812. 10.3389/fendo.2023.1144812 PMC10153672 37143737

[87] Zhang T , Tan T , Han L , et al. Predicting breast cancer types on and beyond molecular level in a multi‐modal fashion. NPJ Breast Cancer. 2023;9(1):16. 36949047 10.1038/s41523-023-00517-2 PMC10033710

[88] Zhang M , Wang C , Cai L , et al. Developing a weakly supervised deep learning framework for breast cancer diagnosis with HR status based on mammography images. Comput Struct Biotechnol J. 2023;22:17‐26. 37655162 10.1016/j.csbj.2023.08.012 PMC10465855

[89] Fan M , Wang K , Pan D , et al. Radiomic analysis reveals diverse prognostic and molecular insights into the response of breast cancer to neoadjuvant chemotherapy: a multicohort study. J Transl Med. 2024;22(1):637. 38978099 10.1186/s12967-024-05487-y PMC11232151

[90] Zhao J , Sun Z , Yu Y , et al. Radiomic and clinical data integration using machine learning predict the efficacy of anti‐PD‐1 antibodies‐based combinational treatment in advanced breast cancer: a multicentered study. J Immunother Cancer. 2023;11(5). 10.1136/jitc-2022-006514 PMC10230987 37217246

[91] Zhang Y , You C , Pei Y , et al. Integration of radiogenomic features for early prediction of pathological complete response in patients with triple‐negative breast cancer and identification of potential therapeutic targets. J Transl Med. 2022;20(1):256. 35672824 10.1186/s12967-022-03452-1 PMC9171937

[92] Zhou J , Bai Y , Zhang Y , et al. A preoperative radiogenomic model based on quantitative heterogeneity for predicting outcomes in triple‐negative breast cancer patients who underwent neoadjuvant chemotherapy. Cancer Imaging. 2024;24(1):98. 39080809 10.1186/s40644-024-00746-z PMC11289960

[93] Lai J , Chen Z , Liu J , et al. A radiogenomic multimodal and whole‐transcriptome sequencing for preoperative prediction of axillary lymph node metastasis and drug therapeutic response in breast cancer: a retrospective, machine learning and international multicohort study. Int J Surg. 2024;110(4):2162‐2177. 38215256 10.1097/JS9.0000000000001082 PMC11019980

[94] Lu N , Guan X , Zhu J , Li Y , Zhang J . A contrast‐enhanced CT‐based deep learning system for preoperative prediction of colorectal cancer staging and RAS mutation. Cancers. 2023;15(18):4497. 37760468 10.3390/cancers15184497 PMC10526233

[95] Cao W , Hu H , Guo J , et al. CT‐based deep learning model for the prediction of DNA mismatch repair deficient colorectal cancer: a diagnostic study. J Transl Med. 2023;21(1):214. 36949511 10.1186/s12967-023-04023-8 PMC10035255

[96] Lee H , Moon SH , Hong JY , Lee J , Hyun SH . A machine learning approach using FDG PET‐based radiomics for prediction of tumor mutational burden and prognosis in stage IV colorectal cancer. Cancers. 2023;15(15):3841. 37568657 10.3390/cancers15153841 PMC10416826

[97] Saber R , Henault D , Messaoudi N , et al. Radiomics using computed tomography to predict CD73 expression and prognosis of colorectal cancer liver metastases. J Transl Med. 2023;21(1):507. 37501197 10.1186/s12967-023-04175-7 PMC10375693

[98] Shi R , Chen W , Yang B , et al. Prediction of KRAS, NRAS and BRAF status in colorectal cancer patients with liver metastasis using a deep artificial neural network based on radiomics and semantic features. Am J Cancer Res. 2020;10(12):4513‐4526. 33415015 PMC7783758

[99] Zhong ME , Duan X , li Ni‐jia‐tiMyidi , et al. CT‐based radiogenomic analysis dissects intratumor heterogeneity and predicts prognosis of colorectal cancer: a multi‐institutional retrospective study. J Transl Med. 2022;20(1):574. 36482390 10.1186/s12967-022-03788-8 PMC9730572

[100] Gao J , Ye F , Han F , Jiang H , Zhang J . A radiogenomics biomarker based on immunological heterogeneity for non‐invasive prognosis of renal clear cell carcinoma. Front Immunol. 2022;13. 10.3389/fimmu.2022.956679 PMC9513051 36177018

[101] He H , Xie Y , Song F , Feng Z , Rong P . Radiogenomic analysis based on lipid metabolism‐related subset for non‐invasive prediction for prognosis of renal clear cell carcinoma. Eur J Radiol. 2024;175:111433. 38554673 10.1016/j.ejrad.2024.111433

[102] Zeng H , Chen L , Wang M , Luo Y , Huang Y , Ma X . Integrative radiogenomics analysis for predicting molecular features and survival in clear cell renal cell carcinoma. Aging. 2021;13(7):9960‐9975. 33795526 10.18632/aging.202752 PMC8064160

[103] Morrison G , Buckley J , Ostrow D , et al. Non‐invasive profiling of advanced prostate cancer via multi‐parametric liquid biopsy and radiomic analysis. Int J Mol Sci. 2022;23(5):2571. 35269713 10.3390/ijms23052571 PMC8910093

[104] Ogbonnaya CN , Alsaedi BSO , Alhussaini AJ , et al. Radiogenomics map‐based molecular and imaging phenotypical characterization in localised prostate cancer using pre‐biopsy biparametric MR imaging. Int J Mol Sci. 2024;25(10). 10.3390/ijms25105379 PMC11121591 38791417

[105] Louis DN , Perry A , Wesseling P , et al. The 2021 WHO classification of tumors of the central nervous system: a summary. Neuro‐Oncol. 2021;23(8):1231‐1251. 34185076 10.1093/neuonc/noab106 PMC8328013

[106] Weller M , Wen PY , Chang SM , et al. Glioma. Nat Rev Dis Primer. 2024;10(1):33. 10.1038/s41572-024-00516-y 38724526

[107] Pellerino A , Caccese M , Padovan M , Cerretti G , Lombardi G . Epidemiology, risk factors, and prognostic factors of gliomas. Clin Transl Imaging. 2022;10(5):467‐475.

[108] Sanai N , Berger MS . Surgical oncology for gliomas: the state of the art. Nat Rev Clin Oncol. 2018;15(2):112‐125. 29158591 10.1038/nrclinonc.2017.171

[109] Gempt J , Förschler A , Buchmann N , et al. Postoperative ischemic changes following resection of newly diagnosed and recurrent gliomas and their clinical relevance. J Neurosurg. 2013;118(4):801‐808. 23373806 10.3171/2012.12.JNS12125

[110] Pei D , Guan F , Hong X , et al. Radiomic features from dynamic susceptibility contrast perfusion‐weighted imaging improve the three‐class prediction of molecular subtypes in patients with adult diffuse gliomas. Eur Radiol. 2023;33(5):3455‐3466. 36853347 10.1007/s00330-023-09459-6

[111] Yoo RE , Yun TJ , Hwang I , et al. Arterial spin labeling perfusion‐weighted imaging aids in prediction of molecular biomarkers and survival in glioblastomas. Eur Radiol. 2020;30(2):1202‐1211. 31468161 10.1007/s00330-019-06379-2

[112] Wei Y , Li C , Cui Z , et al. Structural connectome quantifies tumour invasion and predicts survival in glioblastoma patients. Brain. 2023;146(4):1714‐1727. 36189936 10.1093/brain/awac360 PMC10115235

[113] Karremann M , Gielen GH , Hoffmann M , et al. Diffuse high‐grade gliomas with H3 K27M mutations carry a dismal prognosis independent of tumor location. Neuro‐Oncol. 2018;20(1):123‐131. 29016894 10.1093/neuonc/nox149 PMC5761525

[114] Ryall S , Krishnatry R , Arnoldo A , et al. Targeted detection of genetic alterations reveal the prognostic impact of H3K27M and MAPK pathway aberrations in paediatric thalamic glioma. Acta Neuropathol Commun. 2016;4(1):93. 27577993 10.1186/s40478-016-0353-0 PMC5006436

[115] Pan CC CUN , Liu J , Tang J , et al. A machine learning‐based prediction model of H3K27M mutations in brainstem gliomas using conventional MRI and clinical features. Radiother Oncol. 2019;130:172‐179. 30097251 10.1016/j.radonc.2018.07.011

[116] Su X , Chen N , Sun H , et al. Automated machine learning based on radiomics features predicts H3 K27M mutation in midline gliomas of the brain. Neuro‐Oncol. 2020;22(3):393‐401. 31563963 10.1093/neuonc/noz184 PMC7442326

[117] Esteller M , Garcia‐Foncillas J , Andion E , et al. Inactivation of the DNA‐repair gene MGMT and the clinical response of gliomas to alkylating agents. N Engl J Med. 2000;343(19):1350‐4. 10.1056/NEJM200011093431901 11070098

[118] Li L , Xiao F , Wang S , et al. Preoperative prediction of MGMT promoter methylation in glioblastoma based on multiregional and multi‐sequence MRI radiomics analysis. Sci Rep. 2024;14(1):16031. 38992201 10.1038/s41598-024-66653-2 PMC11239670

[119] Doniselli FM , Pascuzzo R , Mazzi F , et al. Quality assessment of the MRI‐radiomics studies for MGMT promoter methylation prediction in glioma: a systematic review and meta‐analysis. Eur Radiol. 2024;3. 10.1007/s00330-024-10594-x PMC11364578 38308012

[120] Saeed N , Ridzuan M , Alasmawi H , Sobirov I , Yaqub M . MGMT promoter methylation status prediction using MRI scans? An extensive experimental evaluation of deep learning models. Med Image Anal. 2023;90:102989. 37827111 10.1016/j.media.2023.102989

[121] Śledzińska P , Bebyn MG , Furtak J , Kowalewski J , Lewandowska MA . Prognostic and predictive biomarkers in gliomas. Int J Mol Sci. 2021;22(19):10373. 34638714 10.3390/ijms221910373 PMC8508830

[122] Yang K , Wu Z , Zhang H , et al. Glioma targeted therapy: insight into future of molecular approaches. Mol Cancer. 2022;21(1). 10.1186/s12943-022-01513-z PMC8822752 35135556

[123] Haubold J , Hosch R , Parmar V , et al. Fully automated MR based virtual biopsy of cerebral gliomas. Cancers. 2021;13(24). 10.3390/cancers13246186 PMC8699054 34944806

[124] Verduin M , Primakov S , Compter I , et al. Prognostic and predictive value of integrated qualitative and quantitative magnetic resonance imaging analysis in glioblastoma. Cancers. 2021;13(4). 10.3390/cancers13040722 PMC7916478 33578746

[125] Tak D , Ye Z , Zapaischykova A , et al. Noninvasive molecular subtyping of pediatric low‐grade glioma with self‐supervised transfer learning. Radiol Artif Intell. 2024;6(3):e230333. 38446044 10.1148/ryai.230333 PMC11140508

[126] Sun C , Jiang C , Wang X , Ma S , Zhang D , Jia W . MR‐based radiomics predicts CDK6 expression and prognostic value in high‐grade glioma. Acad Radiol. 2024;S1076‐6332(24):00364‐00367. Published online July 3. 10.1016/j.acra.2024.06.006 38964985

[127] Zhao K , Zhang H , Lin J , et al. Radiomic prediction of CCND1 expression levels and prognosis in low‐grade glioma based on magnetic resonance imaging. Acad Radiol. 2024;S1076‐6332(24):00196‐X. Published online. 10.1016/j.acra.2024.03.031 38824087

[128] Hu LS , Ning S , Eschbacher JM , et al. Radiogenomics to characterize regional genetic heterogeneity in glioblastoma. Neuro‐Oncol. 2017;19(1):128‐137. 27502248 10.1093/neuonc/now135 PMC5193022

[129] Xu PF , Li C , Chen YS , et al. Radiomics‐based survival risk stratification of glioblastoma is associated with different genome alteration. Comput Biol Med. 2023;159:106878. 37060774 10.1016/j.compbiomed.2023.106878

[130] Luan J , Zhang D , Liu B , et al. Immune‐related lncrnas signature and radiomics signature predict the prognosis and immune microenvironment of glioblastoma multiforme. J Transl Med. 2024;22(1):107. 38279111 10.1186/s12967-023-04823-y PMC10821572

[131] Hu LS , Hawkins‐Daarud A , Wang L , Li J , Swanson KR . Imaging of intratumoral heterogeneity in high‐grade glioma. Cancer Lett. 2020;477:97‐106. 32112907 10.1016/j.canlet.2020.02.025 PMC7108976

[132] Yoon JH , Kim H . CT radiomics in oncology: insights into the tumor microenvironment of hepatocellular carcinoma. Radiology. 2023;307(1). 10.1148/radiol.222988 36511812

[133] Choi YS , Ahn SS , Chang JH , et al. Machine learning and radiomic phenotyping of lower grade gliomas: improving survival prediction. Eur Radiol. 2020;30(7):3834‐3842. 32162004 10.1007/s00330-020-06737-5

[134] Jang BSS , Park AJ , Jeon SH , et al. Machine learning model to predict pseudoprogression versus progression in glioblastoma using mri: a multi‐institutional study (krog 18‐07). Cancers. 2020;12(9):1‐14. 10.3390/cancers12092706 PMC7564954 32967367

[135] Leiter A , Veluswamy RR , Wisnivesky JP . The global burden of lung cancer: current status and future trends. Nat Rev Clin Oncol. 2023;20(9):624‐639. 37479810 10.1038/s41571-023-00798-3

[136] Thai AA , Solomon BJ , Sequist LV , Gainor JF , Heist RS . Lung cancer. The Lancet. 2021;398(10299):535‐554. 10.1016/S0140-6736(21)00312-3 34273294

[137] Lahiri A , Maji A , Potdar PD , et al. Lung cancer immunotherapy: progress, pitfalls, and promises. Mol Cancer. 2023;22(1):40. 36810079 10.1186/s12943-023-01740-y PMC9942077

[138] Tan AC , Tan DSW . Targeted therapies for lung cancer patients with oncogenic driver molecular alterations. J Clin Oncol. 2022;40(6):611‐625. 34985916 10.1200/JCO.21.01626

[139] Blumenthal GM , Bunn PA , Chaft JE , et al. Current status and future perspectives on neoadjuvant therapy in lung cancer. J Thorac Oncol. 2018;13(12):1818‐1831. 30268698 10.1016/j.jtho.2018.09.017

[140] Arbour KC , Riely GJ . Systemic therapy for locally advanced and metastatic non–small cell lung cancer. JAMA. 2019;322(8):764. 31454018 10.1001/jama.2019.11058

[141] Da Cunha Santos G , Shepherd FA , Tsao MS . EGFR mutations and lung cancer. Annu Rev Pathol Mech Dis. 2011;6(1):49‐69. 10.1146/annurev-pathol-011110-130206 20887192

[142] Blaquier JB , Ortiz‐Cuaran S , Ricciuti B , Mezquita L , Cardona AF , Recondo G . Tackling osimertinib resistance in EGFR‐mutant non‐small cell lung cancer. Clin Cancer Res. 2023;29(18):3579‐3591. 37093192 10.1158/1078-0432.CCR-22-1912

[143] Passaro A , Jänne PA , Mok T , Peters S . Overcoming therapy resistance in EGFR‐mutant lung cancer. Nat Cancer. 2021;2(4):377‐391. 35122001 10.1038/s43018-021-00195-8

[144] Chang C , Zhou S , Yu H , et al. A clinically practical radiomics‐clinical combined model based on PET/CT data and nomogram predicts EGFR mutation in lung adenocarcinoma. Eur Radiol. 2021;31(8):6259‐6268. 33544167 10.1007/s00330-020-07676-x

[145] Yang X , Liu M , Ren Y , et al. Using contrast‐enhanced CT and non‐contrast‐enhanced CT to predict EGFR mutation status in NSCLC patients—a radiomics nomogram analysis. Eur Radiol. 2022;32(4):2693‐2703. 34807270 10.1007/s00330-021-08366-y PMC8921110

[146] Zhao W , Yang J , Ni B , et al. Toward automatic prediction of EGFR mutation status in pulmonary adenocarcinoma with 3D deep learning. Cancer Med. 2019;8(7):3532‐3543. 31074592 10.1002/cam4.2233 PMC6601587

[147] Wang S , Shi J , Ye Z , et al. Predicting EGFR mutation status in lung adenocarcinoma on computed tomography image using deep learning. Eur Respir J. 2019;53(3). 10.1183/13993003.00986-2018 PMC6437603 30635290

[148] Wang S , Yu H , Gan Y , et al. Mining whole‐lung information by artificial intelligence for predicting EGFR genotype and targeted therapy response in lung cancer: a multicohort study. Lancet Digit Health. 2022;4(5):e309‐e319. 35341713 10.1016/S2589-7500(22)00024-3

[149] Schneider JL , Lin JJ , Shaw AT . ALK‐positive lung cancer: a moving target. Nat Cancer. 2023;4(3):330‐343. 36797503 10.1038/s43018-023-00515-0 PMC10754274

[150] Le NQK , Kha QH , Nguyen VH , Chen YC , Cheng SJ , Chen CY . Machine learning‐based radiomics signatures for EGFR and KRAS mutations prediction in non‐small‐cell lung cancer. Int J Mol Sci. 2021;22(17):9254. 34502160 10.3390/ijms22179254 PMC8431041

[151] Mountzios G , Remon J , Hendriks LEL , et al. Immune‐checkpoint inhibition for resectable non‐small‐cell lung cancer — opportunities and challenges. Nat Rev Clin Oncol. 2023;20(10):664‐677. 37488229 10.1038/s41571-023-00794-7

[152] Mu W , Tunali I , Gray JE , Qi J , Schabath MB , Gillies RJ . Abstract 868: prediction of clinical benefit to checkpoint blockade in advanced NSCLC patients using radiomics of PET/CT images. Cancer Res. 2020;80(16_Supplement):868‐868.31772036

[153] Sun R , Limkin EJ , Vakalopoulou M , et al. A radiomics approach to assess tumour‐infiltrating CD8 cells and response to anti‐PD‐1 or anti‐PD‐L1 immunotherapy: an imaging biomarker, retrospective multicohort study. Lancet Oncol. 2018;19(9):1180‐1191. 30120041 10.1016/S1470-2045(18)30413-3

[154] Hinzpeter R , Kulanthaivelu R , Kohan A , et al. Predictive [18F]‐FDG PET/CT‐based radiogenomics modelling of driver gene mutations in non‐small cell lung cancer. Acad Radiol. 2024;S1076‐6332(24):00423‐00429. Published online July 11. 10.1016/j.acra.2024.06.038 38997880

[155] Li L , Duan J , Gao Y , et al. Multi‐omics predictive model based on clinical, radiomic and genomic features for predicting the response of limited‐stage small cell lung cancer to definitive chemoradiotherapy. Clin Transl Med. 2024;14(1):e1522. 38193621 10.1002/ctm2.1522 PMC10775182

[156] Verma S , Magazzù G , Eftekhari N , et al. Cross‐attention enables deep learning on limited omics‐imaging‐clinical data of 130 lung cancer patients. Cell Rep Methods. 2024;4(7):100817. 38981473 10.1016/j.crmeth.2024.100817 PMC11294841

[157] De Miguel‐Perez D , Ak M , Mamindla P , et al. Validation of a multiomic model of plasma extracellular vesicle PD‐L1 and radiomics for prediction of response to immunotherapy in NSCLC. J Exp Clin Cancer Res CR. 2024;43(1):81. 38486328 10.1186/s13046-024-02997-x PMC10941547

[158] Huppert LA , Gumusay O , Idossa D , Rugo HS . Systemic therapy for hormone receptor‐positive/human epidermal growth factor receptor 2‐negative early stage and metastatic breast cancer. CA Cancer J Clin. 2023;73(5):480‐515. 36939293 10.3322/caac.21777

[159] Loibl S , Poortmans P , Morrow M , Denkert C , Curigliano G . Breast cancer. The Lancet. 2021;397(10286):1750‐1769. 10.1016/S0140-6736(20)32381-3 33812473

[160] Acciavatti RJ , Lee SH , Reig B , et al. Beyond breast density: risk measures for breast cancer in multiple imaging modalities. Radiology. 2023;306(3):e222575. 36749212 10.1148/radiol.222575 PMC9968778

[161] Cui H , Sun Y , Zhao D , et al. Radiogenomic analysis of prediction HER2 status in breast cancer by linking ultrasound radiomic feature module with biological functions. J Transl Med. 2023;21(1):44. 36694240 10.1186/s12967-022-03840-7 PMC9875533

[162] Nicosia L , Bozzini AC , Ballerini D , et al. Radiomic features applied to contrast enhancement spectral mammography: possibility to predict breast cancer molecular subtypes in a non‐invasive manner. Int J Mol Sci. 2022;23(23). 10.3390/ijms232315322 PMC9740943 36499648

[163] Bitencourt AGV , Gibbs P , Rossi Saccarelli C , et al. MRI‐based machine learning radiomics can predict HER2 expression level and pathologic response after neoadjuvant therapy in HER2 overexpressing breast cancer. Ebiomedicine. 2020;61:103042. 33039708 10.1016/j.ebiom.2020.103042 PMC7648120

[164] Zhu Z , Albadawy E , Saha A , Zhang J , Harowicz MR , Mazurowski MA . Deep learning for identifying radiogenomic associations in breast cancer. Comput Biol Med. 2019;109:85‐90. 31048129 10.1016/j.compbiomed.2019.04.018 PMC7155381

[165] Huang ZH , Chen L , Sun Y , Liu Q , Hu P . Conditional generative adversarial network driven radiomic prediction of mutation status based on magnetic resonance imaging of breast cancer. J Transl Med. 2024;22(1):226. 38429796 10.1186/s12967-024-05018-9 PMC10908206

[166] Mathews JC , Nadeem S , Levine AJ , Pouryahya M , Deasy JO , Tannenbaum A . Robust and interpretable PAM50 reclassification exhibits survival advantage for myoepithelial and immune phenotypes. Npj Breast Cancer. 2019;5(1):30. 31531391 10.1038/s41523-019-0124-8 PMC6733897

[167] Liang S , Xu S , Zhou S , et al. IMAGGS: a radiogenomic framework for identifying multi‐way associations in breast cancer subtypes. J Genet Genomics Yi Chuan Xue Bao. 2024;51(4):443‐453. 37783335 10.1016/j.jgg.2023.09.010

[168] Gallivanone F , Cava C , Corsi F , Bertoli G , Castiglioni I . In silico approach for the definition of radiomirnomic signatures for breast cancer differential diagnosis. Int J Mol Sci. 2019;20(23). 10.3390/ijms20235825 PMC6929037 31756987

[169] Harris MA , Savas P , Virassamy B , et al. Towards targeting the breast cancer immune microenvironment. Nat Rev Cancer. 2024;24(8):554‐577. 38969810 10.1038/s41568-024-00714-6

[170] Wang H , Ding XH , Liu CL , et al. Genomic alterations affecting tumor‐infiltrating lymphocytes and PD‐L1 expression patterns in triple‐negative breast cancer. J Natl Cancer Inst. 2023;115(12):1586‐1596. 37549066 10.1093/jnci/djad154

[171] Han X , Gong Z , Guo Y , Tang W , Wei X . Exploration of a noninvasive radiomics classifier for breast cancer tumor microenvironment categorization and prognostic outcome prediction. Eur J Radiol. 2024;175:111441. 38537607 10.1016/j.ejrad.2024.111441

[172] Pesapane F , Rotili A , Botta F , et al. Radiomics of MRI for the prediction of the pathological response to neoadjuvant chemotherapy in breast cancer patients: a single referral centre analysis. Cancers. 2021;13(17). 10.3390/cancers13174271 PMC8428336 34503081

[173] Sutton EJ , Onishi N , Fehr DA , et al. A machine learning model that classifies breast cancer pathologic complete response on MRI post‐neoadjuvant chemotherapy. Breast Cancer Res. 2020;22(1):57. 32466777 10.1186/s13058-020-01291-w PMC7254668

[174] Fan M , Chen H , You C , et al. Radiomics of tumor heterogeneity in longitudinal dynamic contrast‐enhanced magnetic resonance imaging for predicting response to neoadjuvant chemotherapy in breast cancer. Front Mol Biosci. 2021;8:622219. 33869279 10.3389/fmolb.2021.622219 PMC8044916

[175] Huang Y , Chen W , Zhang X , et al. Prediction of tumor shrinkage pattern to neoadjuvant chemotherapy using a multiparametric MRI‐based machine learning model in patients with breast cancer. Front Bioeng Biotechnol. 2021;9:662749. 34295877 10.3389/fbioe.2021.662749 PMC8291046

[176] Kocak B , Durmaz ES , Ates E , Ulusan MB . Radiogenomics in clear cell renal cell carcinoma: machine learning–based high‐dimensional quantitative CT texture analysis in predicting PBRM1 mutation status. Am J Roentgenol. 2019;212(3):W55‐W63. 30601030 10.2214/AJR.18.20443

[177] Gao R , Pang J , Lin P , et al. Identification of clear cell renal cell carcinoma subtypes by integrating radiomics and transcriptomics. Heliyon. 2024;10(11):e31816. 38841440 10.1016/j.heliyon.2024.e31816 PMC11152948

[178] Gu J , Zhu J , Qiu Q , Wang Y , Bai T , Yin Y . Prediction of immunohistochemistry of suspected thyroid nodules by use of machine learning‐based radiomics. Am J Roentgenol. 2019;213(6):1348‐1357. 31461321 10.2214/AJR.19.21626

[179] Roy S , Whitehead TD , Quirk JD , et al. Optimal co‐clinical radiomics: sensitivity of radiomic features to tumour volume, image noise and resolution in co‐clinical T1‐weighted and T2‐weighted magnetic resonance imaging. Ebiomedicine. 2020;59:102963. 32891051 10.1016/j.ebiom.2020.102963 PMC7479492

[180] Moher Alsady T , Voskrebenzev A , Behrendt L , et al. Multicenter standardization of phase‐resolved functional lung MRI in patients with suspected chronic thromboembolic pulmonary hypertension. J Magn Reson Imaging. 2024;59(6):1953‐1964. 37732541 10.1002/jmri.28995

[181] Whybra P , Zwanenburg A , Andrearczyk V , et al. The image biomarker standardization initiative: standardized convolutional filters for reproducible radiomics and enhanced clinical insights. Radiology. 2024;310(2):e231319. 38319168 10.1148/radiol.231319 PMC10902595

[182] Varriano G , Guerriero P , Santone A , Mercaldo F , Brunese L . Explainability of radiomics through formal methods. Comput Methods Programs Biomed. 2022;220:106824. 35483269 10.1016/j.cmpb.2022.106824

[183] Liu M , Ning Y , Teixayavong S , et al. A translational perspective towards clinical AI fairness. Npj Digit Med. 2023;6(1):172. 37709945 10.1038/s41746-023-00918-4 PMC10502051

[184] Andaur Navarro CL , Damen JAA , Takada T , et al. Risk of bias in studies on prediction models developed using supervised machine learning techniques: systematic review. BMJ. 2021:n2281. Published online October 20. 34670780 10.1136/bmj.n2281 PMC8527348

[185] Ghassemi M , Oakden‐Rayner L , Beam AL . The false hope of current approaches to explainable artificial intelligence in health care. Lancet Digit Health. 2021;3(11):e745‐e750. 34711379 10.1016/S2589-7500(21)00208-9

[186] Azodi CB , Tang J , Shiu SH . Opening the black box: interpretable machine learning for GenetICIsts. Trends Genet. 2020;36(6):442‐455. 32396837 10.1016/j.tig.2020.03.005

[187] Tomaszewski MR , Gillies RJ . The biological meaning of radiomic features. Radiology. 2021;298(3):505‐516. 33399513 10.1148/radiol.2021202553 PMC7924519

[188] Jiang Y , Zhang Z , Wang W , et al. Biology‐guided deep learning predicts prognosis and cancer immunotherapy response. Nat Commun. 2023;14(1):5135. 37612313 10.1038/s41467-023-40890-x PMC10447467

[189] Liu Q , Hu P . Extendable and explainable deep learning for pan‐cancer radiogenomics research. Curr Opin Chem Biol. 2022;66:102111. 34999476 10.1016/j.cbpa.2021.102111

[190] Zanfardino M , Pane K , Mirabelli P , Salvatore M , Franzese M . TCGA‐TCIA impact on radiogenomics cancer research: a systematic review. Int J Mol Sci. 2019;20(23):6033. 31795520 10.3390/ijms20236033 PMC6929079

[191] Prior F , Smith K , Sharma A , et al. The public cancer radiology imaging collections of The Cancer Imaging Archive. Sci Data. 2017;4(1):170124. 28925987 10.1038/sdata.2017.124 PMC5827108

[192] Ganini C , Amelio I , Bertolo R , et al. Global mapping of cancers: the Cancer Genome Atlas and beyond. Mol Oncol. 2021;15(11):2823‐2840. 34245122 10.1002/1878-0261.13056 PMC8564642

[193] Kirby J , Tarbox L , Freymann J , Jaffe C , Prior F . TU‐AB‐BRA‐03: the cancer imaging archive: supporting radiomic and imaging genomic research with open‐access data sets. Med Phys. 2015;42(6Part31):3587‐3587.

[194] Khalid N , Qayyum A , Bilal M , Al‐Fuqaha A , Qadir J . Privacy‐preserving artificial intelligence in healthcare: techniques and applications. Comput Biol Med. 2023;158:106848. 37044052 10.1016/j.compbiomed.2023.106848

[195] Tang R , Liang H , Guo Y , et al. Pan‐mediastinal neoplasm diagnosis via nationwide federated learning: a multicentre cohort study. Lancet Digit Health. 2023;5(9):e560‐e570. 37625894 10.1016/S2589-7500(23)00106-1

[196] Zhou J , Chen S , Wu Y , et al. PPML‐Omics: a privacy‐preserving federated machine learning method protects patients’ privacy in omic data. Sci Adv. 2024;10(5):eadh8601. 38295178 10.1126/sciadv.adh8601 PMC10830108

[197] Huang P , Li D , Jiao Z , et al. Common feature learning for brain tumor MRI synthesis by context‐aware generative adversarial network. Med Image Anal. 2022;79:102472. 35567847 10.1016/j.media.2022.102472

[198] Ahmadian M , Bodalal Z , van der Hulst HJ , et al. Overcoming data scarcity in radiomics/radiogenomics using synthetic radiomic features. Comput Biol Med. 2024;174:108389. 38593640 10.1016/j.compbiomed.2024.108389

[199] Hosny A , Parmar C , Quackenbush J , Schwartz LH , Aerts HJWL . Artificial intelligence in radiology. Nat Rev Cancer. 2018;18(8):500‐510. 29777175 10.1038/s41568-018-0016-5 PMC6268174

[200] Mitra S . Deep learning with radiogenomics towards personalized management of gliomas. IEEE Rev Biomed Eng. 2023;16:579‐593. 33900921 10.1109/RBME.2021.3075500

[201] Tippareddy C , Onyewadume L , Sloan AE , et al. Novel 3D magnetic resonance fingerprinting radiomics in adult brain tumors: a feasibility study. Eur Radiol. 2023;33(2):836‐844. 35999374 10.1007/s00330-022-09067-w PMC12915495

[202] Wu X , Zhang B . Chatgpt promotes healthcare: current applications and potential challenges. Int J Surg. 2024;110(1):606‐608. 37816164 10.1097/JS9.0000000000000802 PMC10793836

[203] Mehandru N , Miao BY , Almaraz ER , Sushil M , Butte AJ , Alaa A . Evaluating large language models as agents in the clinic. Npj Digit Med. 2024;7(1):84. 38570554 10.1038/s41746-024-01083-y PMC10991271

[204] Bhayana R . Chatbots and large language models in radiology: a practical primer for clinical and research applications. Radiology. 2024;310(1):e232756. 38226883 10.1148/radiol.232756

[205] Li J , Wang S , Zhang M , et al. Agent Hospital: A Simulacrum of Hospital with Evolvable Medical Agents. Published online May 5, 2024.

[206] Hsu JBK , Lee GA , Chang TH , et al. Radiomic immunophenotyping of GSEA‐assessed immunophenotypes of glioblastoma and its implications for prognosis: a feasibility study. Cancers. 2020;12(10). 10.3390/cancers12103039 PMC7603270 33086550

